# Development of a multicomponent survey of experiences of tragedy-based and fear-based trauma in COVID-19 healthcare professionals

**DOI:** 10.3389/fpsyg.2025.1513067

**Published:** 2026-01-27

**Authors:** Paul Gilbert, Marcela Matos, Jaskaran Basran, Kelly Morter, Ptarmigan Plowright, Ana Ferreira, Maria do Céu Salvador, William Kirby

**Affiliations:** 1 Centre for Compassion Research and Training, College of Science and Engineering, University of Derby, Derby, United Kingdom; 2 The Compassionate Mind Foundation, Markeaton Lodge, c/o University of Derby, Derby, United Kingdom; 3 Faculty of Psychology and Educational Sciences, Center for Research in Neuropsychology and Cognitive and Behavioral Intervention (CINEICC), University of Coimbra, Coimbra, Portugal; 4 Royal London Hospital, London, United Kingdom

**Keywords:** anger, anxiety, compassion, COVID-19, empathy, sadness, tragedy, trauma

## Abstract

**Background:**

Life confronts us with many aversive events, including injuries, diseases, losses, decay and death. These life realities often stimulate compassion motives orientated to try to alleviate and prevent suffering. While concepts of ‘stress’ and ‘fear’ have dominated the discourse on responses to ‘traumatic events’, a less common narrative is that of ‘tragedy’. Tragedy narratives focus on the empathic sensitivities to the ‘suffering and traumas of life’ and hence are related to compassion motives. Tragedy has a long history with complex meanings and is focused on sadness and loss, rather than fear. Hence, it invites a different language of experiences requiring grief work rather than (just) fear exposure work. While the experiences of healthcare professionals (HCPs) working with COVID-19 patients have been studied regarding stresses and fear-based traumas, HCPs were also witness to, and experienced these traumas, in terms of tragedy. This study developed a multicomponent survey to explore different dimensions and patterns of HCPs’ experiences with a focus on issues of sadness, grief and tragedy. Focusing on the *tragic* elements of a trauma invites a different narration, language and way of working through trauma.

**Methods:**

From informal discussions with colleagues working in high impact COVID-19 environments, such as intensive care units, and with psychologists who supported those staff, we identified a non-exclusive and non-exhaustive set of themes that textured their experience. We were particularly interested in experiences that could be seen as descriptions of tragedy-based trauma. These were presented as novel self-report surveys to HCPs in British and Portuguese samples. This sought to explore diverse patterns of experiences and responses beyond diagnostic criteria like posttraumatic stress disorder (PTSD). Measures of social safeness, trauma, posttraumatic growth and burnout were also given to explore these themes using standard scales.

**Results:**

Our survey suggested key compassion themes of: high levels of empathic distress for the suffering of others and being more fearful of passing the virus to friends and close others than being infected oneself. As suggested by a tragedy focus, sadness and tearfulness were as prevalent as fear. The compassionate support of others, i.e., family, friends and colleagues, were central for coping. Reflecting on how they had changed over time, many HCPs noted personal growth. In terms of their emotions, the strongest ones HCPs wanted help with were ‘finding joy’, indicating perhaps that loss of textures of positive affect can be a consequence of these events, as in ‘sadder but wiser’ sentiments. These data indicate that the impacts of these types of events are richly textured and extend beyond issues of threat and PTSD symptom-focused approaches. It invites clinicians to explore and contextualise some trauma experiences as tragedy, which facilitates a different languaging and processing of such events.

**Conclusion:**

The way we use language and narrate traumatic events can have a major impact on how we come to process and make sense of them, and how we can help people going through these experiences. This paper suggests that narrating certain types of loss-based trauma events, particularly those linked to health and death (as in COVID-19) can be regarded as forms of human tragedy, which links to more ancient ways of addressing life’s suffering, including the importance of shared compassion and communal grieving. This is an initial exploration of potential experiences of HCPs that can distinguish concepts of tragedy-based trauma from fear-based trauma. Clarity on these variations can offer opportunities for new insights into sources of distress, and therapeutic interventions.

## Introduction

### Exploring trauma and tragedy

While concepts of ‘stress’, ‘fear’ and ‘burnout’ have dominated the discourse on responses to ‘traumatic events’ like COVID-19 (Annaloro et al., 2021; Kira et al., 2021; Lis et al., 2020; Marvaldi et al., 2021), a less common narrative is that of ‘tragedy’. Baran et al.’s (2023) qualitative study explored the working experiences of intensive care unit (ICU) nurses during the pandemic and highlighted different themes relating to relationships, compassion and fear. However, less was revealed in terms of their own emotional responses to the realities of the deaths and the process of dying; that is, their own empathic grief to these ‘life tragedies of the losses’. This is important because of the known link between compassionate engagement with patients and vulnerability to empathic distress and coping with grief in certain clinical areas (Behan and Kelly, 2025). Although depression has been identified as one of the experiences of HCPs (Kira et al., 2021), it is important to distinguish sadness and grieving from depression because they are very different (Harris and Ho, 2023; Horwitz and Wakefield, 2007).

The narrative and definitions of tragedy have a different history, different meanings, and invite exploration of stress-traumatic experiences in new ways to those currently focused on ‘fear-based trauma’. Some years ago, one of the authors (PG) noted that when he empathically reflected on some clients’ experiences with the term *tragedy* rather than trauma, the client typically shifted into focusing on grief, a sense of loss and common humanity. Moreover, those who seemed blocked in their ability to process grief, which could interfere with the therapeutic journey, seemed to be more open to it with discussions about tragedy. These were anecdotal observations, linked to concepts of compassion but with no clear data (Gilbert, 2009; Gilbert, 2022a, pp. 337–340).

Currently, the distinctions and overlaps between fear-focused *vs* tragedy-focused trauma are rarely articulated. For example, the last 30 years has seen considerable research into the causes and experiences of, and therapies for, people who have been through significant threatening life events and traumas (Gold, 2017). Trauma can be defined in terms of an event, the psychophysiological reactions to the event(s) or context, and their interaction. The American Psychological Association (2024) offer the following definition:

*Trauma is an emotional response to a terrible event like an accident, crime, natural disaster, physical or emotional abuse, neglect, experiencing or witnessing violence, death of a loved one, war, and more. Immediately after the event, shock and denial are typical. Longer term reactions include unpredictable emotions, flashbacks, strained relationships, and even physical symptoms like headaches or nausea.*

Problematic in these areas is that terms such as ‘stress,’ ‘trauma’ and ‘disorder’ are not without controversies and ‘fuzzy’ boundaries (Richter-Levin and Sandi, 2021; Richter-Levin et al., 2019). Distinctions regarding ‘a disorder’ have been made on the basis of whether stressors promote *resilience,* both psychologically and biologically, or dysfunctional change, with the emergence of unique symptoms (Richter-Levin and Sandi, 2021). Using resilience as a distinction can be tricky however, because people’s resilience can emerge over time and become a source of growth (Tedeschi and Calhoun, 1996). Hence, a number of researchers have highlighted that both the definitions of a trauma as an event, as distinct from a stressor, and as a disorder distinct from a ‘normal’ reaction, are complex (Richter-Levin and Sandi, 2021; North et al., 2016). North et al. (2016) plotted some of the changes and problems in the history of the American Diagnostic and Statistical Manual of Mental Disorders (DSM-5; APA, 2013) and note that “the PTSD criterion requires not just the occurrence of a traumatic event, but also a qualifying exposure to it” (p. 206). Key to trauma is what the actual threat is. Harris and Machado (2017) note that stressful events can become traumatic partly because of the way they disrupt our experience of the world and throw us into states of uncertainty and threat vigilance:

*Traumatic loss may, in fact, be the violent death of a family member, but it may also be any loss that significantly undermines one’s sense of safety, or that stretches the boundaries of one’s assumptions about how the world should work to the point that there are profound feelings of senselessness, meaninglessness, helplessness, powerlessness, loss of control, and distress (p. 277).*

Although there is some agreement around three major symptom clusters underpinning PTSD: hyperarousal and vigilance, avoidance and numbing, and flashbacks-re-experiencing, which are core to a diagnosis (Grey, 2009; North et al., 2016), it is also recognised that the impact and experience of trauma are highly heterogeneous and require different therapeutic interventions. Traumatic events can shatter people’s beliefs about the world and their ability to deal with it, particularly in terms of it becoming more unpredictable and frightening. Trauma implies injury to one or more of: the physical-body, self-identity, sense of safety and social trust. Hence, traditional approaches to PTSD tend to focus on different types of injury and fear of injury. In contrast, tragedy tends to be focused on sadness and grief. PTSD is also associated with complex unique patterns of emotions such as (for example) anger, anxiety, sadness/grief and disgust. Some individuals can feel intensely rageful while for others, the dominant emotions may be ones of fear or grief/sadness (Behan and Kelly, 2025). Some can experience mood shifts such as anhedonia and numbing (Eskelund et al., 2018), or hopelessness and defeat, which are different to grief states (Siddaway et al., 2015). Traumas can also be linked to self-blame, shame, shame memories and guilt (Lee et al., 2001; Pinto-Gouveia and Matos, 2011) that can impact a long-term sense of one’s identity (Lee, 2022; Matos et al., 2020). These variations can depend on the nature and context of the trauma, personal history and supports for coping.

However, there remain major research gaps exploring the impact of ‘traumatic events’. In a review of the neuroscience of trauma, Fonzo (2018) notes that, “there remains a stark imbalance in the degree to which the neuroscience of each affective domain has been probed and characterised in PTSD, with our knowledge of post-trauma diminished positive affect remaining comparatively underdeveloped” (p. 214). Seidemann et al. (2021) also highlights that “to date, the majority of PTSD research has focused on the inhibition system—fear learning, maintenance, and extinction—due to the conspicuous nature of the disorder’s fear-related symptoms. Though less researched, PTSD also involves depressive symptoms, such as anhedonia and emotional numbing (which relate to the reward system)” (p. 1). This is partly because research on trauma emerged from studies of veteran trauma that became a template for trauma studies, but this might not be a good template for other types of trauma, particularly those linked to the compassion motivation (Gilbert, 2022a, pp. 337–340).

Norcross and Wampold (2019) have also raised concerns about how we define and use terms like trauma and, in particular, how we can turn what can be ‘normal’ reactions to abnormal events into biomedical-type pathologies and disorders, e.g., PTSD (see also Harris and Machado, 2017; Horwitz and Wakefield, 2007; Lee, 2018). They note that an over-focus on such diagnostic approaches risks limiting people’s experiences to a set of ‘diagnostic core symptoms’ and ignoring the multiplicity of experiences and their therapeutic engagement. Indeed, whether mental health difficulties, such as certain depressions and PTSD, should be seen as disorders, reactions or postures has a long history (Hill, 1968) and has invited political critique (Lee, 2018; Harris and Machado, 2017; Horwitz and Wakefield, 2007). It is also a theme central to evolutionary approaches to mental health difficulties (Gilbert, 1989/2016, 2006; Nesse, 2019). Crucial, too, is the degree to which people experience trauma alone and in isolation such as various forms of violence or abuse, in contrast to collective trauma such as from slavery, war, famine, natural disasters and pandemics. All these concerns relate to how contexts influence how people conceptualise, narrate and give voice to life events and their impacts on them. One person may experience a trauma as a test by God (Jung and Pauli, 1952; Jung, 1969) while another as an example of the harshness, meaninglessness and callousness of life (Gilbert, 1989/2016, 2009; Felski, 2008). These conceptualisations impact scientific exploration and clinical engagement.

### Caring, compassion and trauma

The variety of experiences of trauma can have many sources and causes. These can include: non-social versus social, intentional versus unintentional, direct versus vicarious. While most people will deliberately avoid harmful stressors and traumas, there are some circumstances and occupations where individuals deliberately put themselves at risk to help others, such as in the military, police, rescue services, and care and medical services (Fraess-Phillips et al., 2017). Given that compassion motivation is defined as ‘a sensitivity to suffering in self and others with a commitment to try to alleviate and prevent it’ (Gilbert, 2014), compassion motivation is at the heart of those who deliberately and intentionally move out of their own comfort zones, with personal risk, to help others in states of suffering (Gilbert, 2009, 2017, 2020; Ricard, 2015; Seppälä et al., 2017). In a study of COVID-19 public health care, Bhuiyan (2022) found mixed reasons for helping that included: ‘altruistic behaviour, service to profession, adherence to bureaucratic accountability, and a desire to help mankind’ (p. 269).

### Secondary traumatic stress (STS)

Deliberate engagement with suffering exposes people to potential trauma experiences, from risks of *physical* injury faced by the police, military and rescue services, for example, and infections and contamination faced by healthcare workers. In addition, the *emotional* experiences of witnessing the suffering of others, and efforts to help them, is also a source of mental distress. Hence, compassion is very different to kindness, and unlike kindness, requires courage, wisdom and commitment, and with some awareness of potential risk to self (Gilbert, 2020; Gilbert et al., 2019; Kirby et al., 2022). The mental distress arising from engagement with the distress-suffering of others has sometimes been labelled as secondary traumatic stress (STS; Kalaitzaki et al., 2022) and also, depending on context and circumstances, moral trauma (Litam and Balkin, 2021).

In regard to STS, frontline COVID-19 HCPs suffered many adverse reactions indicating high threat arousal, such as anxiety, depression, insomnia, somatisation and symptoms of PTSD (Conti et al., 2020; D’Ettorre et al., 2021; Wild et al., 2022) and burnout distress (Annaloro et al., 2021; Barello et al., 2020). Greene et al. (2021) surveyed 1,194 health and social care workers in May–July 2020, of which 47% met diagnostic criteria for anxiety and depression and 22% for PTSD. Marvaldi et al.’s (2021) meta-analysis estimated 20.2% of frontline HCPs suffered with PTSD symptoms and 31.1% experienced depression. Greenberg et al. (2021) found that 13% of frontline professionals reported suicidal ideation and self-harm. Newman et al. (2022) used social media to survey 395 NHS staff in April through to May, 2020. Their open-ended interviews generated three core themes: (1) Despair and uncertainty (feeling overwhelmed trying to protect everyone); (2) Behavioural and psychological impacts (affecting wellbeing and functioning) and (3) Coping and employer support (getting the right help). They conclude by saying [NHS staff] “felt enormous burden to adequately complete their professional, personal and civil responsibility to keep everyone safe leading to negative psychological and behavioural consequences and desire to receive better support from the NHS employers.” (p. 789). Another source of distress was empathic distress arising from the ‘tragedies’ of the deaths, the impact on their families and the way that HCPs often had to stand in for families and priests (Lake et al., 2022). Clearly then, the level of stress and distress for those engaging in this kind of compassionate action is high.

### Moral trauma and injury

Linked to STS is moral trauma and moral injury (Fischer et al., 2022) and moral distress (Lake et al., 2022). Here, again, there can be elements of tragedy. Litam and Balkin (2021) give the following definition:

Moral injury is the psychological distress that results from actions, or the lack thereof, that violates someone’s moral or ethical code (Greenberg et al., 2020; Litz et al., 2009. Experiences of moral injury are characterized by feelings of shame, guilt, and disgust and negative thoughts about themselves, others, or the world (Greenberg et al., 2020). The compounding effects of making challenging medical decisions, working under extreme pressure, and balancing their own physical and mental health with the needs of their patients, family, and friends are believed to contribute to moral injury in health care professionals during the COVID-19 pandemic (Greenberg et al., 2020; Williamson et al., 2020) (p. 1).

Hence, moral distress can arise when individuals feel they acted against, or have failed to act in line with, their moral principles and values that are key to their sense of self-identity (Fischer et al., 2022; Kip et al., 2022; Mottaghi et al., 2020; Browne et al., 2015). It can also relate to *empathic distress* from being moved by the tragedies of the dying patients, their families and having to act as stand-ins (Lake et al., 2022). Aiken et al. (2023) conducted a large-scale survey involving 21,050 physicians and nurses from US hospitals. Over 40% of respondents said they lacked confidence in management. They also selected management interventions (to improve the delivery of care to patients) as more important to their own mental health and well-being than interventions directly targeting clinicians’ mental health, implicating the importance of adequate patient care for *their own* wellbeing.

Among the crucial traumas and stresses that arise from compassion motivation is what happens if our efforts fail – if the people we are trying to help, be they ‘the rescued’, patients, relatives, or friends, continue to suffer and in some cases, worsen or die. Indeed, Williamson et al. (2020) indicated moral trauma to be especially noted when the people one is trying to help, die. This was a common experience in some COVID-19 care contexts. Although there is scant research on the impact of the death of those one is trying to help or save on those trying to help, the data so far suggests it can be a traumatising experience with many symptoms associated with PTSD (Masia et al., 2010).

Midwives can be confronted by many tragedies, e.g., baby health problems, deformities, stillbirth or subsequent death, threats to the mother and post-birth complications. Linetsky et al. (2024) found that in midwives the rate and intensity of PTSD symptoms were associated with self-criticism, lower self-compassion and pain catastrophising involving ruminating, magnifying and a sense of helplessness. Grief was not explored, but when their patients suffered despite the carer’s best efforts, there could be complex mixtures of self-blame, doubt, guilt, anxiety, anger and grief, depending on the circumstances (Behan and Kelly, 2025). Moral trauma can be linked to elevated levels of self-blame and self-criticism. These problems have been found in COVID-19 HCPs (Zerach and Levi-Belz, 2022). Elevated levels of self-criticism are associated with mental health difficulties (Werner et al., 2019). Importantly, it is the harshness and what is called self-attacking forms of self-criticism (Gilbert et al., 2004) that are particularly problematic (Gilbert, 2022b; Whelton and Greenberg, 2005).

Blaming external factors is also prominent in moral trauma. During COVID-19, HCPs faced limited resources that constantly confronted them with what could or should be possible with what was possible (Greenberg et al., 2020; Litam and Balkin, 2021). Being sensitive to suffering without being able to act as one would like to can lead to more distress (Gilbert et al., 2017; Di Bello et al., 2021). Hence, these stresses were linked to a sense of guilt and shame that one had not been able to care in the way that one would have liked to care. In addition, these were significant stressors in having to constantly step in as a proxy relative at the point of dying, again with worries of how one had been able to act as such. Although some of the ‘symptoms’ linked to STS and moral trauma have been associated with burnout or compassion fatigue, Litam and Balkin (2021) did not find evidence for compassion fatigue. Indeed, it is important to distinguish a genuine loss of motivation (for example for people you do not like) in contrast to wanting to be compassionate but feeling overwhelmed and helpless. However, again this research did not explore empathic grief, although it is common in certain clinical areas (Behan and Kelly, 2025).

As with other pandemics before it, the advent of COVID-19 created multiple arenas for fears, traumas and tragedies in the general population and with the HCPs trying to help. However these efforts were contextualised in multiple sources of stress that included: the nature of the disease, the number of people requiring help, the intense struggles to try to keep people alive with aided respiratory systems and other means, the displacement of other patients from their wards or places of care, the stresses of wearing appropriate protective clothing, not being able to facilitate relative contact with dying patients, and anger with the system in which HCPs were working. What was different in this pandemic to those that had come before was the extraordinary advance in medical technologies that enabled HCPs to save lives, but which also put major stresses on those delivering such services. As a number of studies have noted, unfamiliarity with the technologies and concerns about lacking the skills necessary was a major stress, and in some ways the more ‘miraculous’ medicine becomes (e.g., complex lifesaving surgery) the more HCPs can worry if they are keeping up in regards to their own competencies.

### Tragedy: sadness and grief as dimensions of trauma

A dimension of trauma research that we think is relatively absent is the focus on the experience of *tragedy* rather than (just) fear, secondary traumatic stress and moral trauma. Further, we propose that understanding the psychology of the nature of *tragedy* as rooted in how we process certain types of loss, including the inevitabilities of disease and death, and including those associated with failed efforts to alleviate suffering, can advance understanding and help us to work with certain forms of trauma.

All biological life forms decay and die, and there is evidence that many species will try to help conspecifics who are injured. Some species of ant will even carry injured ants back to the colony (Kessler, 2020). Indeed, one of the evolutionary challenges that may well have driven human caring behaviour was concern and care for the sick and injured (Spikins, 2015). Caring for the sick and injured has always carried risks, not only of diverting resources away from self-care but also because bacterial and viral infections are dangerous and have driven species to extinction. The European bubonic plague (1346–53) killed more than 50 million, over 50% of the population at the time. More people died as a result of the Spanish flu of 1918 than the First World War (National Archives and Records Administration, n.d.).

The point is that part of human existence is being confronted with the normal processes of disease, decay and death but also the extraordinary mass killings from natural disasters and pandemics. Moreover, with heightened human capacities for compassion comes a capacity for empathic grief and sadness for the losses of others. Rahmani et al. (2023) reported that during and after COVID-19, HCPs were at a high risk of suffering from complicated grief (grief that significantly affects daily life for over 1 year; Marie Curie, 2024). Tragedy is a useful concept therefore because most dictionary definitions of tragedy define it as an event of great loss associated with sadness, sorrow and grief. While trauma is associated with fear, threat and numbing (Seidemann et al., 2021), some traumas are associated with intense emotions such as sadness and states of grieving (Gilbert, 2022a, 2023; Harris and Ho, 2023). However, just as the role of positive affect in trauma is understudied, so is the role of sadness and grief. Indeed, Horwitz and Wakefield (2007) brought a number of authors together to highlight how sadness has been gradually edged out of ‘normal human experience’ and can be become seen as forms of depressive disorders, which it is not. While there has been a lot of grief work in close relationships (Gruppi et al., 2022; Harris and Ho, 2023) there is also a long history of philosophical traditions that focus on sadness and grief in relation to life’s suffering, conceptualised as tragedy. *Tragedy* offers a different focus for conceptualising and narrating traumatic events. For example, we tend to focus on the experience of tragedy when thinking about suffering that has befallen *another* as opposed to ‘self-tragedy.’ This aspect of tragedy invites compassion via the experience of empathic distress; we feel distressed by the distress of the one experiencing the life difficulty, loss or misfortune. Indeed, it is empathic sharing that was essential to the Greek dramas. The importance of narrating experiences of (compassion-associated) traumatic events as also ones of tragedy are not currently conceptualised in trauma research or therapies. The efforts to heal moral trauma can involve working with forgiveness and compassion (Litz et al., 2009) but have not been considered in terms of tragedies of evolved human brains that can behave in the harmful ways that they do (Gilbert, 1989/2016, 2009).

### Tragedy: a history of shared experience

The word tragedy has a different origin to trauma, and its meaning and textured experience is more elusive (Conversi and Sewall, 2025; Felski, 2008; Poole, 2005; Steiner, 2004). The Greek origin of tragedy is from *tragos*, meaning ‘goat’ and ‘ōidē’ meaning song (Poole, 2005). The link to its contemporary meaning is unknown, but it might have been associated with the singing of sad songs and laments, with ‘goat’ relating to sacrifice. It became a term to describe a certain type of theatre and drama and it was seen as a reflection on and response to the ordeals of life, as in the Greek tragedies (Felski, 2008; Nietzsche, 1872/2008; Poole, 2005). In theatre, the fates of Romeo and Juliet or Achilles are presented as tragedy rather than trauma. Grief and tragedy vary with meaning. For example, the death of a 90-year-old grandmother from dementia will be seen very differently from the death of a teenager in a car accident or the death of Romeo and Juliet – tragedy conveys a sense of loss of *what could* have been.

Felski (2008) and Poole (2005) discuss how the concept of tragedy was central to many western philosophers such as Plato, Aristotle, Kant, Hegel and Schopenhauer, and the more recent existential movement. Many of these philosophers contextualised the inevitable hardships and suffering of life and its ending (death, including the death of loved ones) as tragedies that all must face. Much of our lives can be spent trying to avoid processing these realities or coming to terms with them. This has been linked to terror management (Juhl and Routledge, 2016). Goldenberg et al. (1999) considered an explanation for why we are attracted to storylines of tragedy that are sometimes regarded as ‘tearjerkers’ like the Greek tragedies, because they can touch a part of us that is aware that ‘all that we love and all that we are, will pass away and be lost’, i.e., ‘all is impermanent’, as is stressed in the contemplative traditions (Anālayo, 2015). They found that priming people with this kind of morality awareness made them more sensitive and emotionally moved by tragedy scenarios. Key to tragedy is *grief* and *sense of unfairness* (Felski, 2008). Although sadness is discussed in the diagnosis of PTSD, it is linked to depression (Seidemann et al., 2021), that can disengage it from normal, intense grieving (Horwitz and Wakefield, 2007). Grief and sense of unfairness are also rarely discussed, but for pandemics and disasters, the arbitrariness of suffering can itself be traumatic and spiritually challenging. In the context of caring, HCPs can grieve for themselves (e.g., the losses of their own life disruptions) but also they can suffer immense empathic grief for the death and suffering of their patients and relatives.

How we narrate people’s distressing life experiences in terms of trauma and/or tragedy will impact our engagement and training. For example, Cook et al. (2019) explored many important dimensions for training HCPs in ‘sensitivity to trauma’ yet the concept of tragedy and the importance of guiding people through grief is less explored. Most therapies for trauma focus on exposure and desensitisation of some kind, although some trauma therapists also highlight that for some clients, grief can be profound and requires careful attention, analysis and time (Ogden et al., 2006) especially with its many varieties (Gilbert, 2022a; Harris and Ho, 2023). One dimension is grief for the life one has lost or has been robbed of by the trauma and its effects (Gilbert, 2023).

Grief work is central to compassion focused therapy and plays a major role in working with shame and the sense of disconnection that shame creates, which compounds trauma experiences (Gilbert, 2022b; Harris and Ho, 2023). Over a number of years, one of the authors (PG) has observed that narrating certain distressing life events in terms of tragedy-based trauma can lead more naturally into explorations of grief, whereas narrating them as trauma tends to create a more fear-based and damaged focus. An area where these differences may be especially important is when intense losses emerge within caring relationships, such as how clinical staff encountered COVID-19. As of June 2025, the World Health Organisation (WHO, 2025) reports 7.1 million deaths from COVID-19, and it is estimated to have led to excess deaths as high as 27 million (Our World in Data, 2025).

### The compassion of others

There is now considerable evidence that the quality of our social relationships plays a huge role in the biopsychosocial processing of threat and trauma events, impacting the autonomic nervous system, numerous neurocircuits, hormones and immune system functions (Slavich et al., 2023). Hence, the social contexts in which threats appear is crucial to their impact and subsequent effects. There is a large literature on the importance of the emotional and practical helpfulness and support of others in dealing with life crises (Taylor, 2011). Having a compassionate orientation to self and others has been shown to be a major coping strategy for stress with a variety of physiological effects (Singer, 2025). In a large multinational study of 4,057 adults across 21 countries using the Compassionate Engagement and Action scales (Gilbert et al., 2017), Matos et al. (2022) found that:

*Perceived threat of COVID-19 was associated with higher scores in depression, anxiety, and stress, and lower scores in social safeness. Self-compassion and compassion from others were associated with lower psychological distress and higher social safeness. Compassion for others was associated with lower depressive symptoms. Self-compassion moderated the relationship between perceived threat of COVID-19 on depression, anxiety, and stress, whereas compassion from others moderated the effects of fears of contracting COVID-19 on social safeness.*

Behan and Kelly (2025) have highlighted how compassion engagement automatically gives rise to empathic distress and grief in the face of suffering and dying and the need to develop appropriate supports for working through grief. Miotto et al. (2020) highlighted the importance of providing emotional support for HCPs. It is important that care and support can come from the individual’s own support system in terms of friends and family, and from their organisation. Feelings of safety and safeness have profound effects on a range of physiological systems (Gilbert, 2024; Slavich et al., 2023). These will texture people’s abilities to cope with the tragedies that are unfolding around them. One of the complications is that fear of infecting family, friends and relatives might have reduced intimacy between people. Hence, understanding how people can recruit their personal networks to provide a secure base and safe haven and how these texture the experiences confronting them is an important research area.

### Overview

**Figure 1:**
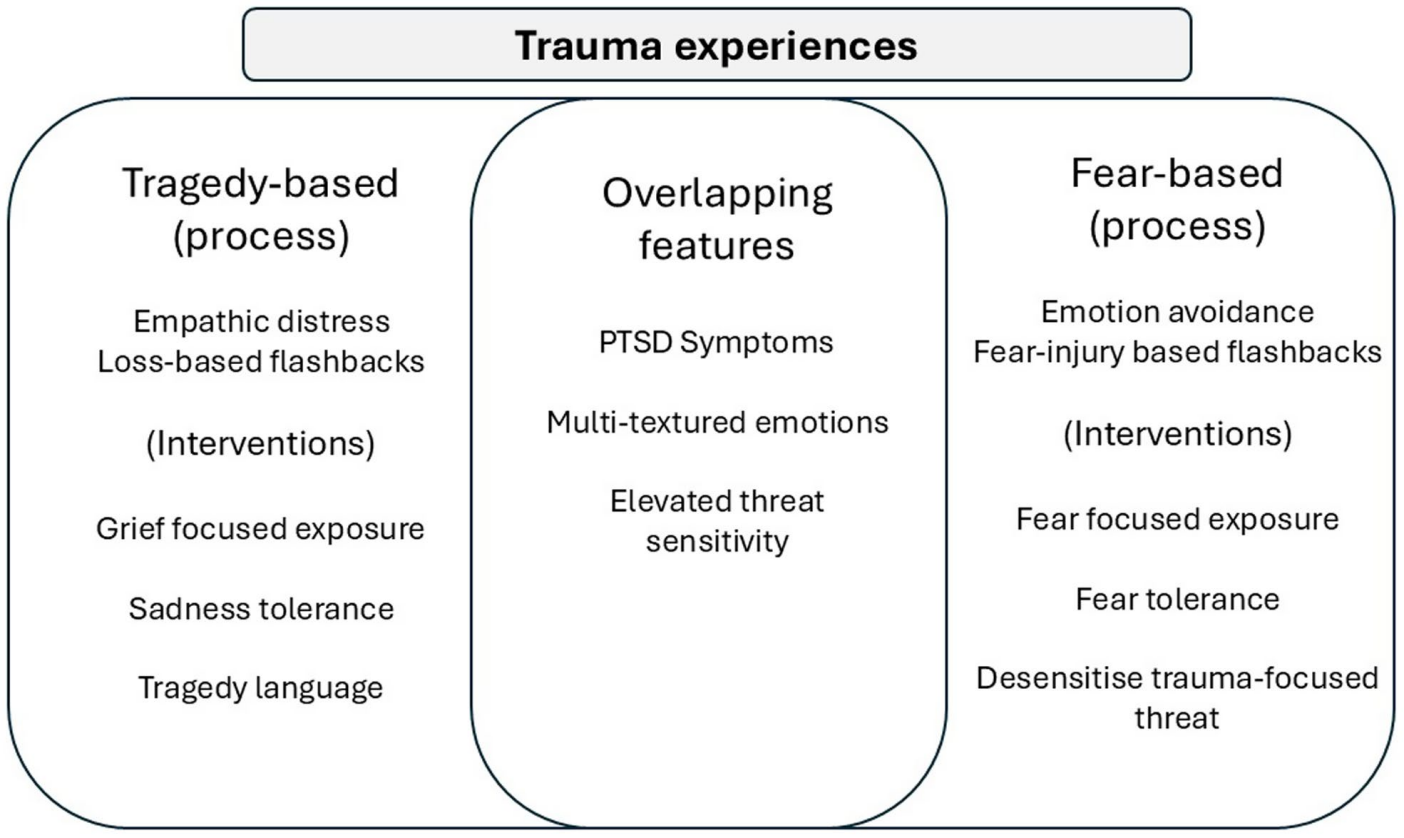
Variations in trauma experience.

As highlighted above, there is general agreement that experiences of trauma can vary vastly in terms of the patterns of emotional responses and focus (Norcross and Wampold, 2019). Within this heterogeneity, we suggest that the concept of tragedy, which is loss and grief-focused, can be distinguished from fear and injury-focused experiences, and that most traumas will have some combination to different degrees. For example, working through the trauma from abusive parenting may also require grieving for the ‘archetypal’ parent that children evolved to need and yearn for (Gilbert, 2023). We also suggest that in some kinds of contexts, such as compassionate efforts to help others (as for COVID-19 HCPs), empathic distress at the suffering of others can be experienced and narrated in terms of tragedy. We further suggest that fear-based aspects of trauma and tragedy-based aspects of trauma require different therapeutic interventions. Moreover, compassion focused interventions are particularly suited to address the tragedy aspects of trauma (Lee, 2022). Figure 1 offers an overview of how trauma experiences can be textured in different ways according to whether the focus is on a tragedy or a fear-based process, with recognition that there are many overlapping features. These variations can also indicate different patterns of intervention.

### Aims

Many studies have explored the high levels of stress and trauma experienced by HCPs in relation to COVID-19 (e.g., Greene et al., 2021; Lamb et al., 2021; Duarte et al., 2022; Jordan et al., 2023; Newman et al., 2022). However, at the time of developing our study in 2020, some of these studies were unavailable. Hence, we explored the same themes from our discussions with colleagues and staff working in these areas. We wanted to explore a multi-dimensional and multi-textured set of experiences in terms of severity and perceived change. In addition, we wanted to contextualise the research in terms of the flows of compassion; that is the experiences people had in regard to their own mental states, the experiences that arose from compassion to others (patients) and the effects of compassionate support and help from others.

As Norcross and Wampold (2019) indicate, current trauma measures are focused on diagnosis rather than capturing the multi-textured experiences associated with the traumas and tragedies of life. This study, therefore, sought to develop a multicomponent survey that tapped into experiences of HCPs treating COVID-19 patients, such as those working in Intensive Care Units (ICUs). Rather than developing specific questionnaires, we wanted to measure *patterns of experience.* We were interested in whether such a multi-textured approach would enable us to detect patterns linking to how people would experience tragedy-based and fear-based trauma.

## Methods

### Procedure

This was a cross-sectional, survey-based design. We began planning and developing the study in the October of 2021 and received ethical permission in the UK in March 2022 from the Health, Psychology and Social Care Research Ethics Committee at the University of Derby. Data were gathered for the UK sample between April 2022 and January 2023. The survey and measures were completed via Qualtrics (www.qualtrics.com, Qualtrics, Provo, UT). Participants were provided with an information sheet and then gave their consent before filling in the surveys. They were provided a debriefing sheet at the end.

Initially, the UK team had hoped to collect data from ICU units within an NHS Trust. However, due to administrative concerns and staff changes, we instead collaborated with ICU charities to collect data. These charities provide community hubs and support to ICU HCPs, hence ethical approval took longer than anticipated and data was gathered over a longer period. Thus, after March 2022, when the UK research team started collecting data, almost all COVID-19 restrictions had been lifted and rates of hospital admission had fallen substantially (Office for National Statistics, 2022), allowing useful insight into the change in HCPs’ experiences. Despite the lifting of most restrictions within that time, there was large strain on the NHS,^1^ the removal of social distancing measures increased stress levels in HCPs and there were worries about COVID-19 returning in the winter of 2023.

In Portugal, the research team translated the scales for use in the Portuguese sample, and the back translations were examined by a bilingual researcher for accuracy and fidelity to the original scales. After ethical approval was granted by the Ethics Committee of the Faculty of Psychology and Educational Sciences at the University of Coimbra in November 2021, the research team contacted clinical boards of national general hospitals and health centers, inviting units directly involved in the treatment of COVID-19 patients to collaborate in the study (e.g., ICUs, Internal Medicine, Pneumonology, Emergency). The healthcare institutions that agreed to collaborate disseminated the study (via email) to the clinical boards of all the health units directly involved in the treatment of COVID-19 patients and respective HCPs. Data were gathered from the Portuguese sample between November 2021 and April 2022 in an online format, through the University’s Institutional LimeSurvey platform. Before completing the self-report questionnaires, participants provided their informed consent. Contact details for the researchers were made available if participants needed any clarification, and psychological support lines were also provided in case participants experienced distress during or after the study.

### Participants

HCPs working closely with COVID-19 patients were recruited via email and completed the questionnaires using the online survey tools detailed above.

#### UK sample

Participants were recruited using invitations sent to ICU charities’ email lists. The final sample comprised 85 adults aged 24–68 years (*M* = 43.06; *SD* = 11.48). Within this sample, 84.7% (*n* = 72) reported an ICU as their normal workplace with a mean of 9 years’ experience. The mean number of years since qualified was 17. Of all the HCPs, 65% reported they had previously contracted COVID-19 (*n* = 55). 34% (*n* = 29) lost colleagues to COVID-19. Participants reported that March to July 2020 was when conditions were most stressful.

#### Portuguese sample

The final sample included 106 adults aged 22–66 years (*M* = 41.61; *SD* = 11.04). Of these HCPs, 11.3% (*n* = 12) reported the ICU as their normal place of work with a mean of 10 years’ ICU experience. The mean number of years since qualified was 16. Of the whole sample, 39% (*n* = 41) reported they had previously contracted COVID-19. 8% (*n* = 8) had lost colleagues to COVID-19. Participants were asked when working conditions were most stressful. 43.4% (*n* = 46) reported that, since the beginning of the pandemic, there was more than one particularly stressful time, 18.9% (*n* = 20) reported the period of March to June 2020 was most stressful, 13.2% (*n* = 14) reported the period of January to March 2021, and 17% (*n* = 18) reported that conditions were always stressful.

Given the UK sample was almost purely ICU HCPs’, whereas the Portuguese sample was linked to a community sample working with COVID-19 patients, we provide the data for each population separately.

### Survey methodology

As this is a preliminary exploration of a complex array of varied experiences in the context of caring for COVID patients, we chose to use the survey method. Ponto (2015) offers a good summary, noting that:

Survey research is defined as “the collection of information from a sample of individuals through their responses to questions” (Check and Schutt, 2012, p. 160). This type of research allows for a variety of methods to recruit participants, collect data, and utilize various methods of instrumentation. Survey research can use quantitative research strategies (e.g. using questionnaires with numerically rated items), qualitative research strategies (e.g. using open-ended questions), or both strategies (i.e. mixed methods). As it is often used to describe and explore human behaviour, surveys are therefore frequently used in social and psychological research (p. 168).

### Measures

#### Multicomponent survey of experiences of tragedy-based and fear-based trauma

The researchers designed this survey in collaboration with clinical staff working in COVID-19 areas and ICU wards including areas with high death rates (see Supplementary material for full survey). We tried to capture common experiences that our informal and open discussions with these HCPs suggested. For example, one of the authors is a specialist respiratory physiotherapist at a major London teaching hospital who worked in an ICU with the most critically ill. He was also part of a support group where the stresses of COVID-19 were discussed.

The questionnaire and instructions are given below with two examples of response format:

“We have set out a series of questions to capture some of your common experiences. This focuses on different flows and domains of caring behaviour relating to the personal experiences of stress, the distress that was triggered through empathic engagement with patients and the experiences of the potential helpfulness of others including the support or stress from the organisation. These questions ask about the thoughts and feelings you may have had in your COVID-19 caring role. Please rate the items using the following rating scale in terms of how much the question applies to you.”

**Table:** 

1	2	3	4	5	6	7
Not at all	Moderately					Extremely

##### Did/do

Each question offers you an opportunity to note the degree to which you did experience what is suggested in the question and if you still do experience it. This will allow us to see how things may have changed for you. An example of self-focused distress is the first question, and an example of empathic distress is the second question.

##### Fear distress

Feel anxious about getting COVID-19 yourself and its impact on you personally?

**Table:** 

*Did*	1	2	3	4	5	6	7
*Do*	1	2	3	4	5	6	7

##### Empathic distress

Empathy is when we imagine ourselves in another person’s situation and feel with them. To what extent did/do you become distressed yourself because of what was happening to your patients due to COVID-19?

**Table:** 

*Did*	1	2	3	4	5	6	7
*Do*	1	2	3	4	5	6	7

The scale was developed in two parts. Part 1 asks participants brief demographic questions and information about their work and family environment during COVID-19. Part 2 of the scale has 5 sections. Section 1 explores psychological and physical reactions in the work; section 2 explores thoughts and feelings around the organisation they have worked in; section 3 explores relationships with others; section 4 explores flashbacks and what support has been helpful, and section 5 explores personal changes including how participants have changed as a result of the COVID-19 crisis. Participants are asked to rate each statement from 1 (‘not at all’) to 7 (‘extremely’). For most questions, participants are asked to reflect on the degree to which they *did* have the experience suggested in the question and if they still *do* experience it. In other words, they were reflecting on their experience over time, of *then* and *now*. This explores reflections on how their mental states may have changed for them. Four open-ended questions are also posed, such as: ‘what was the most difficult aspect of dealing with people outside of your working environment?’

Participants were asked to complete the following questionnaires once, and responses related to the time at which they filled in the questions.

#### Social safeness and pleasure scale

The SSPS (Gilbert et al., 2009) is an 11-item measure that assesses the frequency with which individuals feel a sense of warmth, safeness and reassurance in their social relationships at the time of questionnaire completion. Using a scale from 1 to 5, participants rate items such as: “I feel a sense of belonging,” “I feel secure and wanted,” and “I feel accepted by people.” The scale shows strong construct and discriminant validity and a high degree of reliability (Gilbert et al., 2009; Kelly et al., 2012) with a Cronbach’s alpha above 0.9 (Basran et al., 2019; Kelly and Dupasquier, 2016).

#### Impact of event scale–revised

The IES-R (Weiss and Marmar, 1997) is a 22-item scale which assesses subjective distress caused by traumatic events within the week leading up to questionnaire completion. Each item is rated on a 5-point scale using anchors between 0 (not at all) and 4 (extremely), reflecting the extent to which a statement was a problem for them during the past week. These instructions were adapted slightly to mention COVID-19. The IES-R total score ranges from 0 to 88 and subscale scores can also be calculated for the intrusion, avoidance and numbing, and hyperarousal subscales. These subscales show strong internal consistency (intrusion Cronbach’s *α* = 0.9; avoidance and numbing Cronbach’s α = 0.86; hyperarousal Cronbach’s α = 0.85; Beck et al., 2008). The overall scale demonstrates high reliability with a Cronbach’s α of 0.95. There are many studies supporting the validity of the scale (Asukai et al., 2002; King et al., 2009; Sveen et al., 2010).

#### The posttraumatic growth inventory

This 21-item self-report measure assesses positive outcomes reported by people who have experienced traumatic events (Tedeschi and Calhoun, 1996). Items include “I changed my priorities about what is important in life.” This instrument is organized into five subscales that represent Relating to Others, New Possibilities, Personal Strength, Spiritual Change and Appreciation of Life. Participants are asked to rate on a 6-point Likert scale how much they experienced the changes described by each item, from 0 (‘I did not experience this change as a result of my crisis’) to 5 (‘I experienced this change to a very great degree as a result of my crisis’). Tedeschi and Calhoun (1996) reported Cronbach’s alpha’s of 0.84 (New Possibilities); 0.85 (Relating to Others); 0.72 (Personal Strength); 0.85 (Spiritual Change) and 0.67 (Appreciation of Life). Internal consistency for the whole scale is excellent (Cronbach’s α = 0.90). The Portuguese sample in the present study did not complete the PTGI.

#### Shirom-Melamed burnout measure

Originally comprising 22 items (Kushnir and Melamed, 1992), the Shirom–Melamed Burnout Questionnaire (SMBQ) was revised to form the 14-item SMBM (Lerman et al., 1999) used in this study. The SMBM consists of the three subscales labelled “physical fatigue” (six items, including: “I feel physically drained” and “I feel fed-up”), “cognitive weariness” (five items, including: “I feel I am not thinking clearly” and “I have difficulty concentrating”) and “emotional exhaustion” (three items, e.g., “I feel I am unable to be sensitive to the needs of coworkers and customers”). The scale was adapted to reflect ‘others’ rather than ‘co-workers and customers’. Participants rated on a 7-point Likert scale from 1 (‘almost never’) to 7 (‘almost always’) how often in the last month they have ‘felt this way’ in their job. Shoman et al. (2023) reported moderate internal consistency for the scale although some psychometric properties were found to be insufficient.

### Open-ended questions

Participants were also asked four (voluntary) open-ended questions such as “What was the most difficult aspect of dealing with people outside of your working environment?” and “What did you find helpful?.” These were included to allow participants to express any additional thoughts they may have had or which came up for them during the survey.

### Data analysis

All quantitative data was analysed using SPSS version 28. We experienced delays with accessing the data due to issues with the data gathering program being updated. The data was screened for normality using parameters of skewness and kurtosis. Although all values were within the cut-off points of 2 for skewness and 7 for kurtosis (West et al., 1995), some variables had a non-normal distribution as shown by the histograms. Given that statistical methods to normalise data distribution (e.g., log transformations, square root transformations) can lead to biased estimates and difficulties of interpretation (McCuen et al., 1990; Lee, 2020), and can substantially impact Pearson correlations, we chose to use nonparametric Spearman’s rank correlations to explore the relationships between variables.

As the multicomponent survey of experiences of tragedy-based and fear-based trauma used single item questions, frequency analysis was conducted. Ratings on this survey were grouped into ‘low scores’ (1, 2, 3) and ‘medium to high scores’ (4, 5, 6, 7). Mean imputation is recommended for small amounts of ‘missing completely at random’ data (Tabachnick and Fidell, 2007) and performs as well as other imputation techniques (Peyre et al., 2011; Shrive et al., 2006). For this study, nine participants in the UK sample had a small amount of data (one or two data points) missing, which were replaced with the mean score. Only one participant in the UK sample had over 25% missing data in a section of the survey, so their data for that section was removed. A sensitivity analysis for missing data was conducted but it had no impact on results (Leurent et al., 2018). There was no missing (quantitative) data for the Portuguese sample. There were no outliers on more than one scale, therefore none were removed. Independent samples *t*-tests and Mann–Whitney tests showed significant differences between the UK and Portuguese data for many items, thus data from the two samples were analysed separately and compared.

Descriptives (means and standard deviations), reliability analyses and Spearman’s rank correlations were conducted on suitable study data. Data gathered from four voluntary open-ended questions was reviewed although it was not suitable for in-depth qualitative analysis due to the questions’ concise nature, and because not all participants provided answers. Instead, we briefly discuss the main findings in prose in the following section.

## Results

Although survey data can be subjected to factor analysis, we were uncertain if this would be appropriate given the wide variety of experiences that the survey was assessing, both current experiences (*do*) and retrospective ones (*did*). Preliminary exploration suggested no clear structure and that many of the items would be excluded based on their communalities scores. We therefore did not proceed with the analysis further and instead present it as items of individual experiences. Descriptive statistics (means and standard deviations) and Cronbach’s alpha’s of the other study measures are provided in the tables below.

Below we offer a series of tables that provide frequency data regarding the key questions of our multicomponent survey. English data is presented in italics below the Portuguese data in the tables. Data was gathered during different time periods for the respective samples.

As this is an item-by-item measure, item responses are not presented in their original order. Rather, we categorised and clustered responses according to overarching themes. As the data was collected at one time and we were asking HCPs to reflect on how they felt when things were at their worst and how they felt at the time of data collection, this allowed us to observe change according to their own personal reflections. Hence the responses are given as “did” (at the time of the worst point) and “do” (at the time of participation).

We have clustered the responses into the following core themes according to sections:

Section 1: *Self-focused distress*. This reports responses regarding personal experiences such as anxiety, depression and flashbacks.
Section 2: *Distress for others’ distress*. This reports responses regarding the distress HCPs were experiencing on behalf of their patients: a type of empathic distress.
Section 3: *Openness to the compassion and helpfulness from others*. This reports on responses relating to how helpful others were and are.
Section 4: *Organisational stress*. This reports on responses of how HCPs experience their organisation.
Section 5: *Personal change*. This reports on responses of how people thought their work with COVID-19 patients had changed them.

### Section 1: self-focused distress

#### Primary emotions

As noted above, many studies have indicated a multiple and varied range of different emotions and experiences associated with COVID-19 activity. The questions in Table 1 explored HCP’s four core threat-focused emotional patterns towards the pandemic itself.

**Table 1:** Primary emotions.

*Primary emotions.* To what extent did/do the emotions below describe your primary feelings towards the pandemic?					
Items		Percentage (frequencies)		*M*	*SD*
		Low scores (1–3)	Medium to high scores (4–7)		
Anxiety	*Did*	17 (18/106) *16.5 (14/85)*	83 (88/106) *83.5 (71/85)*	5.2 *5.4*	1.76 *1.6*
	*Do*	52.8 (56/106) *45.9 (39/85)*	47.2 (50/106) *54.1 (46/85)*	3.56 *3.93*	1.86 *1.83*
Anger	*Did*	56.6 (60/106) *48.2 (41/85)*	43.4 (46/106) *51.8 (44/85)*	3.19* 3.84*	2.15 *1.84*
	*Do*	75.5 (80/106) *69.4 (59/85)*	24.5 (26/106) *30.6 (26/85)*	2.27* 2.94*	1.75 *1.62*
Sadness	*Did*	21.7 (23/106) *14.1 (12/85)*	78.3 (83/106) *85.9 (73/85)*	5 *5.31*	1.76 *1.57*
	*Do*	46.2 (49/106) *41.2 (35/85)*	53.8 (57/106) *58.8 (50/85)*	3.59 *4.05*	1.83 *1.85*
Depression	*Did*	57.5 (61/106) *54.1 (46/85)*	42.5 (45/106) *45.9 (39/85)*	3.24 *3.73*	2.1 *1.82*
	*Do*	70.8 (75/106) *68.2 (58/85)*	29.2 (31/106) *31.8 (27/85)*	2.51* 3.08*	1.8 *1.61*

Top is Portuguese sample. Below italics is UK sample.

*Indicates a significant difference between the Portuguese and UK data on that item, according to an independent samples *t*-test and Mann–Whitney test.

#### Anxiety

Over 80% of respondents reflected on their anxiety as being at moderate to high intensity in the early stages of the pandemic. Reflecting on it at the time of data collection, anxiety had reduced, but remained elevated at around 50% suggesting ongoing anxiety.

#### Anger

There were many newspaper reports that clinical staff were angry because they felt the government could have done more to provide them with appropriate personal protective equipment (PPE) and were also angry about staff shortages (Newman et al., 2022). Here anger was rated at 43–52%.

#### Sadness

Given the nature of the traumatic events HCPs were dealing with, it is not surprising sadness was nearly as high as anxiety in the Portuguese sample and higher than anxiety in the UK sample, with 78–86% rating it as moderate-high. While sadness does not feature prominently in clinical discourses on COVID-19, it is a core emotion associated with ‘tragedy’.

#### Depression

Although we did not make any attempt to define depression, less than 50% recalled feeling this mood state. However, at the time of the study, still around 30% were feeling depressed, which warrants further investigations as to how long and for how many these mood states last.

#### Flashbacks

Flashbacks are defining characteristics of PTSD and many studies have suggested these are common in COVID-19 HCPs. We suspected that tragedy and the issue of sadness dominated HCPs experience of these traumatic events as much as fear, therefore we wanted to explore the frequency and emotional textures of flashbacks, which are shown in Table 2.

**Table 2:** Flashback and the emotions of flashbacks.

Items		Percentage (frequencies)		*M*	*SD*
		Low scores (1–3)	Medium to high scores (4–7)		
*Flashback frequency.* Get flashbacks or sudden switches in your mental state that take you back to those distressing experiences?	*Did*	58.5 (62/106) *58.8 (50/85)*	41.5 (44/106) *41.2 (35/85)*	3.07 *3.48*	2.25 *2.14*
	*Do*	76.4 (81/106) *75.3 (64/85)*	23.6 (25/106) *24.7 (21/85)*	2.28 *2.72*	1.68 *1.68*
*Flashback emotion.* If you did/do have flashbacks, to what extent are they associated with feelings of:					
Sadness and grief?	*Did*	59.4 (63/106) *56.5 (48/85)*	40.6 (43/106) *43.5 (37/85)*	3.04 *3.51*	2.12 *2.17*
	*Do*	75.5 (80/106) *71.8 (61/85)*	24.5 (26/106) *28.2 (24/85)*	2.38 *2.87*	1.72 *1.82*
Anxiety?	*Did*	34.9 (37/106) *36.5 (31/85)*	65.1 (69/106) *63.5 (54/85)*	4.17 *3.95*	2.37 *1.57*
	*Do*	62.3 (66/106) *57.6 (49/85)*	37.7 (40/106) *42.4 (36/85)*	3.06 *3.18*	2.03 *1.6*
Anger?	*Did*	65.1 (69/106) *61.2 (52/85)*	34.9 (37/106) *38.8 (33/85)*	2.87 *3.29*	2.18 *2.01*
	*Do*	79.2 (84/106) *74.1 (63/85)*	20.8 (22/106) *25.9 (22/85)*	2.19* 2.66*	1.67 *1.61*

Top is Portuguese sample. Below italics is UK sample.

*Indicates a significant difference between the Portuguese and UK data on that item, according to an independent samples *t*-test and Mann–Whitney test.

#### Flashback frequency

Although flashbacks are a common experience in PTSD, they also occur in other mental states. Our data suggests that around 40% of HCPs experienced flashbacks at a moderate to high intensity. These reduced over time, although were still above 20% at the time of the study. We cannot distinguish between those in highly intense clinical areas from those in less intense areas, but it is likely that these would relate to the frequency and intensity of flashbacks.

#### Flashback association with sadness, anxiety and anger

Flashbacks come not only with sensory and visual textures, but also different textures of emotion. Hence, we were interested in the varied emotions that texture flashbacks. This data indicates that while anxiety was the common texture of flashbacks, sadness and grief also textured flashbacks in over 40% of HCPs. Over a third also acknowledged flashbacks textured with anger. Again, this data indicates the multiplicity of the emotional textures of flashbacks.

#### Emotional experiences

Table 3 looks at a wider range of emotions and emotional experiences. Again, many of these are indicated in other studies, but these specific questions explore intensity and change.

**Table 3:** A spectrum of experiences.

Items		Percentage (frequencies)		*M*	*SD*
		Low scores (1–3)	Medium to high scores (4–7)		
*Fear distress*. Feel anxious about getting COVID-19 yourself and its impact on you personally?	*Did*	30.2 (32/106) *29.4 (25/85)*	69.8 (74/106) *70.6 (60/85)*	4.42 *4.84*	1.96 *1.79*
	*Do*	70.8 (75/106) *57.6 (49/85)*	29.2 (31/106) *42.4 (36/85)*	2.73* 3.53*	1.75 *1.7*
*Unpredictability distress*. Feel distressed because it was uncertain how long the pressure from the pandemic would last?	*Did*	11.3 (12/106) *8.2 (7/85)*	88.7 (94/106) *91.8 (78/85)*	5.42 *5.73*	1.71 *1.38*
	*Do*	29.2 (31/106) *24.7 (21/85)*	70.8 (75/106) *75.3 (64/85)*	4.41* 4.99*	1.9 *1.81*
*Sleep.* Experience sleep difficulties because of working with COVID-19 patients?	*Did*	46.2 (49/106) *41.2 (35/85)*	53.8 (57/106) *58.8 (50/85)*	3.9 *4.39*	2.31 *2.03*
	*Do*	68.9 (73/106) *60 (51/85)*	31.1 (33/106) *40 (34/85)*	2.68* 3.46*	1.83 *1.71*
*Bad Dreams.* Have difficult or nightmarish dreams?	*Did*	59.4 (63/106) *54.1 (46/85)*	40.6 (43/106) *45.9 (39/85)*	3.13* 3.75*	2.32 *2.04*
	*Do*	77.4 (82/106) *75.3 (64/85)*	22.6 (24/106) *24.7 (21/85)*	2.16* 2.92*	1.69 *1.56*
*Emotional exhaustion.* Find yourself becoming emotionally exhausted?	*Did*	20.8 (22/106) *17.6 (15/85)*	79.2 (84/106) *82.4 (70/85)*	5.14 *5.39*	1.87 *1.71*
	*Do*	35.8 (38/106) *29.4 (25/85)*	64.2 (68/106) *70.6 (60/85)*	4.29 *4.81*	2.07 *1.91*
*Numbness distress.* Find yourself becoming emotionally numb?	*Did*	55.7 (59/106) *44.7 (38/85)*	44.3 (47/106) *55.3 (47/85)*	3.42* 3.99*	2.13 *1.83*
	*Do*	57.5 (61/106) *55.3 (47/85)*	42.5 (45/106) *44.7 (38/85)*	3* 3.64*	1.93 *1.81*
*Physical exhaustion.* Find yourself becoming physically exhausted?	*Did*	15.1 (16/106) *12.9 (11/85)*	84.9 (90/106) *87.1 (74/85)*	5.37 *5.65*	1.7 *1.55*
	*Do*	29.2 (31/106) *25.9 (22/85)*	70.8 (75/106) *74.1 (63/85)*	4.52 *5.01*	1.9 *1.81*
*PPE.* Find wearing PPE distressing and difficult because it was uncomfortable?	*Did*	29.2 (31/106) *28.2 (24/85)*	70.8 (75/106) *71.8 (61/85)*	4.75 *4.91*	2.2 *1.99*
	*Do*	47.2 (50/106) *41.2 (35/85)*	52.8 (56/106) *58.8 (50/85)*	3.66 *4.15*	2.03 *1.84*

Top is Portuguese sample. Below italics is UK sample.

*Indicates a significant difference between the Portuguese and UK data on that item, according to an independent samples *t*-test and Mann–Whitney test.

#### Fear distress

Around 70% of both the British and Portuguese samples expressed moderate to high anxiety regarding contracting COVID-19, although this also means a third were less anxious about contracting the virus. This anxiety decreased over time but more so in the Portuguese population. At the time of the study, more than 40% of the British participants were still anxious about catching COVID-19.

#### Unpredictability distress

Uncertainty about how long the pandemic (and hence the pressures at work) would last, created very high levels of distress. This dropped to 71–75% at the time of the study, but clearly was still high. It is likely to be ignited if new waves and new variants increase infection rates.

#### Sleep

Poor sleep and fatigue can be major problems in these contexts and here we see that around half of HCPs struggled with sleep. This dropped over time, but still 40% of the UK sample were struggling with sleep at the time of the study.

#### Bad dreams

Stressful conditions can create problematic dreams which interfere with restful sleep. Studies have indicated that some staff did experience nightmares. In this study, we find that 41–46% acknowledged they had ‘difficult or nightmarish dreams’. This reduced at the time of the study, but with 23–25% still experiencing moderate-high levels this again indicates that the trajectory of these experiences needs to be better studied.

#### Emotional exhaustion

Because of the long hours and the need for specialist staff, emotional exhaustion was often noted by HCPs. This is confirmed in that around 80% felt emotionally exhausted. Other studies had indicated problems of burnout as a result of emotional exhaustion (Duarte et al., 2022).

#### Numbness distress

When confronted with this level of distress day in and day out, numbness can be an issue for some HCPs. It is often regarded as a symptom of trauma, although here it may also relate to exhaustion. Again, around 50% were experiencing moderate to high numbness. Newman et al. (2022) noted that some HCPs had experiences of feeling ‘unreal’ and possibly slight dissociation.

#### Physical exhaustion

Over 85% felt physically exhausted and this remained high at the time of data collection at 71–74%. A slightly higher percentage of the UK sample felt physically exhausted at both timepoints compared to the Portuguese sample.

#### PPE uncomfortable

Many HCPs noted that wearing heavy gowns, masks and PPE was uncomfortable and problematic as it took time to re-suit, for example if one wanted to use the toilet or take a break.

#### Self-criticism and self-reassurance

When we engage with life difficulties, a crucial process is how we relate to and treat ourselves. Issues of self-criticism in relationship to HCPs working in COVID-19 areas have been noted in the literature. The two questions in Table 4 therefore explored their reflections on the intensity of their harsh self-criticism and the degree to which they could be self-reassuring. These are not bipolar dimensions so people can score themselves as high or low on both.

**Table 4:** Self-criticism and self-reassurance.

*Items*		*Percentage (frequencies)*		*M*	*SD*
		*Low scores (1–3)*	*Medium to high scores (4–7)*		
*Self-criticism*. Find it easy to become harshly self-critical of yourself?	*Did*	40.6 (43/106) *32.9 (28/85)*	59.4 (63/106) *67.1 (57/85)*	4.09 *4.45*	2.11 *2.05*
	*Do*	49.1 (52/106) *42.4 (36/85)*	50.9 (54/106) *57.6 (49/85)*	3.54 *3.92*	1.90 *1.92*
*Self-reassurance.* Find you are able to reassure and support yourself when things are difficult?	*Did*	40.6 (43/106) *37.6 (32/85)*	59.4 (63/106) *62.4 (53/85)*	3.82 *4.27*	1.85 *1.87*
	*Do*	35.8 (38/106) *38.8 (33/85)*	64.2 (68/106) *61.2 (52/85)*	3.99 *4.41*	1.85 *1.79*

Top is Portuguese sample. Below italics is UK sample.

#### Self-criticism

Self-criticism is well known as a major transdiagnostic process. Here we wanted to explore *harsh* self-criticism rather than helpful forms (Gilbert, 2022b). Despite doing amazing work, high numbers of people identified themselves as being harshly self-critical. What is interesting is that this decreases, but not much, over time and may well be a very important area to target when supporting staff in these areas. It is also worth noting that a higher percentage of the UK sample report being harshly self-critical. The reason behind this difference is unknown but it could relate to different organisational pressures.

#### Self-reassurance

Self-reassurance is not the mirror opposite of self-criticism and in fact, people can switch between them, so it is important to see that people were also able to self-reassure. Maybe they were self-criticising and self-reassuring on different processes but the ability to be self-reassuring is important for well-being and coping. Concerning, however, is that up to 40% seem to have struggled with self-reassurance and maybe confidence in their behaviours. This would be a source of stress and requires further investigations.

#### Wanting help

While it is important to explore the multi-textured experiences of HCPs as they engage with distressed and dying patients, it is also important to explore what they would like compassionate help with. Hence, we asked a series of questions about what emotions or experiences they would still want help with. These are presented in Table 5.

**Table 5:** Emotions HCPs want help with.

*Help with*. As a result of what you have been through, to what extent do you find the emotion(s) you most need help with:			*M*	*SD*
*Items*	*Percentage (frequencies)*			
	*Low scores (1–3)*	*Medium to high scores (4–7)*		
are sadness and grief?	68.9 (73/106) *68.2 (58/85)*	31.1 (33/106) *31.8 (27/85)*	2.59 *2.98*	1.73 *1.73*
is fear?	70.8 (75/106) *64.7 (55/85)*	29.2 (31/106) *35.3 (30/85)*	2.60 *3.04*	1.86 *1.82*
is anger?	75.5 (80/106) *74.1 (63/85)*	24.5 (26/106) *25.9 (22/85)*	2.35 *2.64*	1.69 *1.40*
is finding joy?	57.5 (61/106) *50.6 (43/85)*	42.5 (45/106) *49.4 (42/85)*	3.28 *3.68*	2.18 *2.07*

Top is Portuguese sample. Below italics is UK sample.

Interestingly we found that around a third of HCPs would like help with dealing with emotions of sadness and grief, fear and anger but the one that scored most highly was finding joy. It is as if going through this experience may have affected some people’s ability to re-engage with positive emotion which, as noted in our introduction, is a relatively ignored dimension of trauma, particularly in these kinds of traumatic and tragic experiences. This links with Fonzo’s (2018) point that the impact of positive affect on these traumas is under researched.

### Section 2: *Distress for others’ distress*

There are unique characteristics of fear-based *and tragedy-based trauma* when people choose to enter into states of other people’s suffering. These are forms of empathic, other-focused distress. This makes them a very different type of distress-trauma to, say, the trauma of a car accident or violence against the self and one’s own injuries. These are explored in Table 6.

**Table 6:** Experiences relating to engaging with the distress of others (empathic distress).

Items		Percentage (frequencies)		*M*	*SD*
		Low scores (1–3)	Medium to high scores (4–7)		
*Protect friend/family distress*. Feel anxious about getting COVID-19 and then passing it on to others including your friends and family?	*Did*	12.3 (13/106) *10.6 (9/85)*	87.7 (93/106) *89.4 (76/85)*	5.70 *5.78*	1.67 *1.56*
	*Do*	42.5 (45/106) *34.1 (29/85)*	57.5 (61/106) *65.9 (56/85)*	4.07 *4.62*	1.96 *2.01*
*Empathic distress*. Empathy is when we imagine ourselves in another person’s situation and feel with them. To what extent did/do you become distressed yourself because of what was happening to your patients due to COVID-19?	*Did*	12.3 (13/106) *10.6 (9/85)*	87.7 (93/106) *89.4 (76/85)*	5.32 *5.55*	1.45 *1.47*
	*Do*	33 (35/106) *25.9 (22/85)*	67 (71/106) *74.1 (63/85)*	4.35* 4.89*	1.85 *1.82*
*Breathing.* Compared to other patient difficulties you have worked with, to what extent did/do you find the specific problems of breathing difficulties due to COVID-19 in patients distressing?	*Did*	39.6 (42/106) *37.6 (32/85)*	60.4 (64/106) *62.4 (53/85)*	3.92 *4.33*	2.00 *1.89*
	*Do*	55.7 (59/106) *45.9 (39/85)*	44.3 (47/106) *54.1 (46/85)*	3.17* 3.80*	1.80 *1.73*
*(Empathic) Sadness distress*. Experience sadness as a result of what was happening with your patients due to COVID-19?	*Did*	13.2 (14/106) *7.1 (6/85)*	86.8 (92/106) *92.9 (79/85)*	5.35 *5.56*	1.51 *1.40*
	*Do*	40.6 (43/106) *28.2 (24/85)*	59.4 (63/106) *71.8 (61/85)*	4.10* 4.69*	1.79 *1.80*
*Tearful(ness).* Moved to tears as a result of what was happening with your patients due to COVID-19?	*Did*	50.9 (54/106) *47.1 (40/85)*	49.1 (52/106) *52.9 (45/85)*	3.43* 4.13*	2.18 *1.94*
	*Do*	71.7 (76/106) *63.5 (54/85)*	28.3 (30/106) *36.5 (31/85)*	2.39* 3.24*	1.70 *1.70*
*Saving patients.* Find it difficult to come to terms with the fact there were patients you were unable to save due to COVID-19?	*Did*	33 (35/106) *25.9 (22/85)*	67 (71/106) *74.1 (63/85)*	4.45 *4.88*	1.99 *1.83*
	*Do*	51.9 (55/106) *43.5 (37/85)*	48.1 (51/106) *56.5 (48/85)*	3.49* 4.11*	1.91 *1.85*
*Tragedy.* Feel overwhelmed with sadness for the tragedy of pandemic deaths you experienced?	*Did*	37.7 (40/106) *32.9 (28/85)*	62.3 (66/106) *67.1 (57/85)*	4.11 *4.56*	2.06 *1.94*
	*Do*	60.4 (64/106) *51.8 (44/85)*	39.6 (42/106) *48.2 (41/85)*	3.02* 3.66*	1.69 *1.78*
*Responsibility distress*. Feel distressed at having to make life and death decisions?	*Did*	63.2 (67/106) *61.2 (52/85)*	36.8 (39/106) *38.8 (33/85)*	2.89* 3.52*	2.18 *2.00*
	*Do*	74.5 (79/106) *70.6 (60/85)*	25.5 (27/106) *29.4 (25/85)*	2.34* 3.13*	1.88 *1.79*
*Helpless distress*. Feel distressed because you felt/feel unable to prevent people you were caring for from dying or harm?	*Did*	27.4 (29/106) *25.9 (22/85)*	72.6 (77/106) *74.1 (63/85)*	4.46 *4.92*	1.99 *1.83*
	*Do*	47.2 (50/106) *40 (34/85)*	52.8 (56/106) *60 (51/85)*	3.48* 4.09*	1.88 *1.72*
*PPE.* Find wearing PPE difficult due to it interfering with you relating to patients?	*Did*	29.2 (31/106) *25.9 (22/85)*	70.8 (75/106) *74.1 (63/85)*	4.63 *4.86*	2.08 *1.95*
	*Do*	43.4 (46/106) *34.1 (29/85)*	56.6 (60/106) *65.9 (56/85)*	3.91 *4.36*	2 *1.91*
*Anger/frustration distress for others*. Feel angry, irritated or frustrated by equipment shortage for your patients? e.g. ventilators, beds.	*Did*	49.1 (52/106) *43.5 (37/85)*	50.9 (54/106) *56.5 (48/85)*	3.79 *4.12*	2.38 *2.06*
	*Do*	78.3 (83/106) *78.8 (67/85)*	21.7 (23/106) *21.2 (18/85)*	2.18* 2.69*	1.77 *1.52*

Top is Portuguese sample. Below italics is UK sample.

*Indicates a significant difference between the Portuguese and UK data on that item, according to an independent samples *t*-test and Mann–Whitney test.

#### Protect friend/family distress

In both the British and Portuguese samples, concerns with infecting friends and family were high at nearly 90% and higher than fears of self-infection (see below). This indicates that a major source of stress and fear when working with infectious disorders is that of infecting others, especially in this case, as it is a life-threatening condition. Indeed, infecting others from working in COVID-19 areas was one of the highest sources of anxiety. This reduced over time, but was still rated as moderate-high by over 65% of the UK respondents at the time of the study.

#### Empathic distress

Empathic distress explored the degree to which HCPs were tuning into the distress of those they were caring for. In both samples, close to 90% reported moderate to high distress. This reduced marginally to 70% at the time of the study which is still high and that is likely because, although the numbers of people infected and dying of COVID-19 had fallen, there were still people suffering from the disease. Our data indicates that in these contexts, where others are suffering empathic distress, the management of empathic distress needs careful consideration and support. Empathic distress is rarely explored in PTSD studies.

#### Breathing

Anecdotal evidence from our informal discussions suggested that caring for people who are dying because they cannot breathe is particularly distressing and this was acknowledged in over 60% of our respondents. Given, as noted above, HCPs were clearly experiencing empathic distress, then this would be an intense form of it—watching somebody die fighting for breath.

#### (Empathic) sadness distress

Sadness is linked to empathic distress, and both are linked to experiences of tragedy. From our discussions with HCPs, it was not just the process of dying, but also the way that patients were cut off from saying goodbye to relatives and relatives were cut off from saying goodbye to their loved ones, which again strikes at the heart of the experience as being one of tragedy as well as being traumatic. Our data suggests very high prevalence of sadness for the suffering of their patients. Sadness facilitators and inhibitors are important processes of human reactions to these types of traumatic events but are rarely explored in PTSD or facilitated in therapy. However, our data suggests these are crucial experiences and working with grief could be crucial to help some people.

#### Tearful(ness)

Half of the HCPs were *often* moved to tears which corroborates anecdotal evidence that, at times, HCPs went home and cried. We have no data as to whether HCPs shared their tearfulness or whether this tended to be at times when they were alone. As noted below (Table 6), some respondents acknowledged difficulty in sharing feelings. It is possible that the intensity of their sadness and grief might have been a case in point. More data on this is required.

#### Saving patients

Many HCPs (74.1% of the UK sample) found it difficult to come to terms with the high death rates in these clinical areas. There could be many reasons they thought they were unable to save people, for example because of the disease itself, but also due to equipment and staff shortages, or their own skills. Such causal explanations would play a prominent role in people’s reactions and stress coping.

#### Tragedy

As noted above, a high percentage of HCPs experienced sadness. Sadness is the core experience associated with tragedy. However, we also wanted to explore peoples’ experiences when using the word “tragedy” and the degree to which they may have felt overwhelmed by the tragic experiences that people were having to go through. 67.1% of the UK respondents felt a moderate to high sense of being overwhelmed by the tragedy of what was happening to their patients.

#### Responsibility distress

Interestingly, over a third of HCPs experienced responsibility distress. We suspect this number could be higher if the sample had been constrained to those who had to make those life and death decisions. Clearly, in these contexts, painful dilemmas feed into responsibility distress, trauma and tragedy experiences. This theme likely links to experiences of personal responsibility, self-blame and self-reassurance.

#### Helpless distress

Nearly 75% of respondents acknowledged feeling distressed by feelings of helplessness at being unable to prevent deaths. Although this is about personal feelings, we have placed it in this category of empathic distress because it is stimulated by the death of patients.

#### PPE interference with patient relating

Many HCPs noted wearing heavy gowns and masks (PPE) interfered with relating to patients. Anecdotal evidence suggests this is because they could not show facial expressions and masks slightly changed voice quality.

#### Anger/frustration distress for others

HCPs were also frustrated because of the equipment shortage that affected patients directly, such as the availability of beds and ventilators. This improved as the pandemic waned but still over 21% felt angry and frustrated at the time of data collection.

### Section 3: openness to the compassion and helpfulness from others

There is considerable evidence that social relationships, particularly those that provide support and caring, have major impacts on a range of biopsychosocial systems that can impact not only mental states but also immune systems which can play a role in virus susceptibility (Slavich et al., 2023). Hence, over and above practical supports, relational supports are crucial. HCPs, like all of us, can be socially embedded and supported in relations with partners, family, friends, professional colleagues and networks. These can be significant, compassionate resources for coping with stress, recovery and posttraumatic growth. Our questions in Table 7 explored how HCPs experienced this network of relationships to their clinical work. On the other side however, people who are avoidant or fearful of seeking and responding to compassionate support and help can be at a disadvantage (Matos et al., 2021a, 2021b).

**Table 7:** Characteristics of what was found helpful and not helpful, or feared, in relating to others.

Items		Percentage (frequencies)		*M*	*SD*
		Low scores (1–3)	Medium to high scores (4–7)		
*Being understood*. Feel other people can understand what you have been through?	*Did*	57.5 (61/106) *47.1 (40/85)*	42.5 (45/106) *52.9 (45/85)*	3.28 *3.68*	1.63 *1.76*
	*Do*	59.4 (63/106) *52.9 (45/85)*	40.6 (43/106) *47.1 (40/85)*	3.21 *3.61*	1.67 *1.68*
*Family support*. Feel you have family and friends who you can turn to for support and help?	*Did*	16 (17/106) *18.8 (16/85)*	84 (89/106) *81.2 (69/85)*	5.42 *5.42*	1.72 *1.75*
	*Do*	17.9 (19/106) *17.6 (15/85)*	82.1 (87/106) *82.4 (70/85)*	5.42 *5.41*	1.70 *1.69*
*Colleagues support*. Feel you have work colleagues who you can turn to for support and help?	*Did*	22.6 (24/106) *18.8 (16/85)*	77.4 (82/106) *81.2 (69/85)*	4.83 *5.05*	1.71 *1.78*
	*Do*	26.4 (28/106) *23.5 (20/85)*	73.6 (78/106) *76.5 (65/85)*	4.73 *4.85*	1.82 *1.93*
*Openness.* Feel comfortable sharing distressing feelings with others?	*Did*	37.7 (40/106) *36.5 (31/85)*	62.3 (66/106) *63.5 (54/85)*	4.00 *4.25*	1.91 *1.96*
	*Do*	38.7 (41/106) *38.8 (33/85)*	61.3 (65/106) *61.2 (52/85)*	3.84 *4.12*	1.89 *1.85*
*Turning to others.* Feel comfortable turning to others and accepting their help?	*Did*	42.5 (45/106) *41.2 (35/85)*	57.5 (61/106) *58.8 (50/85)*	3.88 *4.13*	1.76 *1.87*
	*Do*	44.3 (47/106) *43.5 (37/85)*	55.7 (59/106) *56.5 (48/85)*	3.89 *4.12*	1.92 *1.87*
*Expectations*. Feel others expect you to cope better with these experiences?	*Did*	43.4 (46/106) *32.9 (28/85)*	56.6 (60/106) *67.1 (57/85)*	3.92* 4.45*	1.97 *1.76*
	*Do*	49.1 (52/106) *38.8 (33/85)*	50.9 (54/106) *61.2 (52/85)*	3.72 *4.25*	2.00 *1.83*
*Avoidance.* Feel other people have moved away from you in fear of getting infected by you?	*Did*	54.7 (58/106) *50.6 (43/85)*	45.3 (48/106) *49.4 (42/85)*	3.33 *3.82*	2.32 *1.99*
	*Do*	82.1 (87/106) *76.5 (65/85)*	17.9 (19/106) *23.5 (20/85)*	2.14* 2.85*	1.69 *1.61*

Top is Portuguese sample. Below italics is UK sample.

*Indicates a significant difference between the Portuguese and UK data on that item, according to an independent samples *t*-test and Mann–Whitney test.

#### Being understood

In regard to feeling understood by others, nearly 60% felt they were not that well understood. It is possible that it was the sadness and grief aspects that were particularly difficult. For example, one colleague who acknowledged being very tearful and could not get a particular patient out of their head was advised not unkindly ‘but it’s your job; you just have to put it behind you, leave it at work’. This may also relate to feelings that for people who were not actually there and experienced all of the complexities of PPE, the high death rates, intense stress and so forth, it is difficult for them to understand. Interestingly, this lack of being understood increased slightly over time, in both samples.

#### Family support

The support of family and friends is clearly important and over 80% of the sample acknowledged that support, but some people felt less supported. The degree of support from the home and friendship environment may be particularly relevant for clinical staff who perhaps were from other parts of the country or overseas and were not connected in a support network.

#### Colleagues support

The importance of support from colleagues is also well noted here. The majority of HCPs felt they could turn to colleagues for support, but again we note that nearly a fifth did not. We are unable to explore the reasons for this, for example whether HCPs had come from other clinical areas and teams and not integrated into their working environments.

#### Openness

Linked to the ability to elicit compassionate and caring support from others is the ability to be open with them about what one feels, rather than concealing. While most people did feel reasonably free to be open, 37–38% seemed to struggle with this. This may also fit with the data for those who did not feel understood. More research on what blocks peoples’ openness about their feelings would be important in supporting them.

#### Turning to others

Turning to others is likely linked to trusting, being understood, colleague support and openness. Again, the data suggests that nearly 50% of respondents did struggle with this.

#### Expectations

There was some indication that some HCPs felt they were expected to cope. The degree to which this is a projection is, of course, unknown, but again this can be a factor in experiencing stress, trauma and tragedy in this environment.

#### Avoidance

From the media and anecdotally, fears that others would become less engaged when they discovered that one was working in a high infection area was not borne out by the data, although a small percentage certainly did feel this. Again, why this variation exists is unclear.

#### Sources of support

Finding out what has been helpful to people as they have gone through these kinds of crises is important because they can then be a source of focus and support. This data is presented in Table 8.

**Table 8:** Experiences of who has been helpful.

What or who has helped you most?				
*Items*	*Percentage (frequencies)*		*M*	*SD*
	*Low scores (1–3)*	*Medium to high scores (4–7)*		
Family	11.3 (12/106) *14.1 (12/85)*	88.7 (94/106) *85.9 (73/85)*	5.79 *5.62*	1.66 *1.61*
Friends	15.1 (16/106) *17.6 (15/85)*	84.9 (90/106) *82.4 (70/85)*	5.23 *5.22*	1.65 *1.74*
Work colleagues	23.6 (25/106) *23.5 (20/85)*	76.4 (81/106) *76.5 (65/85)*	4.75 *4.86*	1.77 *1.91*
My professional training	18.9 (20/106) *17.6 (15/85)*	81.1 (86/106) *82.4 (70/85)*	5.08 *5.13*	1.71 *1.84*
Organisational support	70.8 (75/106) *67.1 (57/85)*	29.2 (31/106) *32.9 (28/85)*	2.70 *2.96*	1.61 *1.69*

Top is Portuguese sample. Below italics is UK sample.

*Indicates a significant difference between the Portuguese and UK data on that item, according to an independent samples *t*-test and Mann–Whitney test.

Table 8 shows that, for the most part, families and colleagues were major sources of support. We cannot distinguish people who were well established in their family and community groups in comparison to people who, for example, had come from overseas or were living alone. Professional training and feeling one has the skills for the challenge was clearly important and helpful. We see that organisational support was only at around a third and most people did not find their organisation particularly supportive. Further research is needed to explore the details of that in order to develop better organisational support systems, e.g., by communicating with staff as to what would be helpful to them and what they feel they would need.

### Section 4: organisational stress

In our informal discussions with HCPs, they noted several organisational stresses which we tried to capture in the questions shown in Table 9.

**Table 9:** Organisational stress.

*Items*		*Percentage (frequencies)*		*M*	*SD*
		*Low scores (1–3)*	*Medium to high scores (4–7)*		
*Familiarity.* Were you familiar with the workings of an intensive care unit?		56.6 (60/106) *60 (51/85)*	43.4 (46/106) *40 (34/85)*	3.19 *3.59*	2.36 *2.08*
*Anxiety over work area*. How anxious were you moving into an intensive care unit?		67 (71/106) *64.7 (55/85)*	33 (35/106) *35.3 (30/85)*	2.64* 3.41*	2.27 *2.08*
*Anger/frustration distress for you*. Feel angry, irritated or frustrated by equipment shortages for you? e.g. PPE	*Did*	44.3 (47/106) *34.1 (29/85)*	55.7 (59/106) *65.9 (56/85)*	4.12 *4.35*	2.24 *1.89*
	*Do*	84 (89/106) *77.6 (66/85)*	16 (17/106) *22.4 (19/85)*	2.00* 2.61*	1.46 *1.30*
*Management.* Feel distressed by the way your service was managed?	*Did*	22.6 (24/106) *18.8 (16/85)*	77.4 (82/106) *81.2 (69/85)*	4.85 *5.22*	1.96 *1.71*
	*Do*	44.3 (47/106) *40 (34/85)*	55.7 (59/106) *60 (51/85)*	3.84 *4.29*	2.13 *1.90*
*Shift distress.* Feel distressed because of shift patterns?	*Did*	57.5 (61/106) *51.8 (44/85)*	42.5 (45/106) *48.2 (41/85)*	3.39* 4.02*	2.32 *2.05*
	*Do*	63.2 (67/106) *60 (51/85)*	36.8 (39/106) *40 (34/85)*	2.87* 3.66*	2.18 *1.95*
*Psychological support.* Feel your organisation provided you with psychological support to help you through the crisis?	*Did*	75.5 (80/106) *70.6 (60/85)*	24.5 (26/106) *29.4 (25/85)*	2.42* 3.06*	1.90 *1.75*
	*Do*	78.3 (83/106) *70.6 (60/85)*	21.7 (23/106) *29.4 (25/85)*	2.33* 3.01*	1.89 *1.72*

Top is Portuguese sample. Below italics is UK sample.

*Indicates a significant difference between the Portuguese and UK data on that item, according to an independent samples *t*-test and Mann–Whitney test.

The context in which people work, over and above the nature of the work, can also be a source of support or stress and trauma (Dutton et al., 2014). Anecdotal evidence from our informal discussions suggested this was something HCPs felt strongly about. It is important to keep in mind that the HCPs who participated in this study were from diverse groups and some will have been more frontline and intensively involved than others.

#### Familiarity

From informal discussions, some HCPs were moved from one clinical area into the COVID-19 areas and were not that familiar with that intensity of working. In fact, over 50% acknowledged low familiarity which is represented in our next question on anxiety.

#### Anxiety over work area

This reveals there were high levels of anxiety associated with moving into an ICU, particularly if the HCP was not used to working in one. Again, we have no data on how that was managed, but one can imagine that would contribute to high stress levels; this may well pertain to working with new team members and lacking confidence in these clinical areas.

#### Anger/frustration distress for you

Informal discussions and news media reports at the height of the pandemic suggested some frustration with shortages that impacted HCPs’ ability to do their jobs and keep safe, such as with PPE. This is reflected in the data, with 50% being in the higher endorsement of frustration range.

#### Management

In various healthcare studies, management is often a source of stress and annoyance, and it appears this was no different in the COVID-19 working areas where around 80% felt distressed by the way the service was managed. Informal discussions suggested lack of support, communication and consultation were irritations.

#### Shift distress

Many HCPs were asked to change shift patterns or work extra shifts because of staff shortages, urgencies and emergencies. Shift distress affected just under half of the sample.

#### Psychological support

Organisations do have responsibilities for providing emotional and psychological support to their HCPs. However, nearly 75% of respondents did not rate the availability of this support highly. Interestingly, it does not seem to have improved over time.

### Section 5: personal change

There is increasing interest in how people come through these life experiences and are changed by them for better or for worse. Crucial can be the feeling of making a meaningful contribution that increases our sense of self-efficacy and value to others. Also, coming through trauma helps us recognise that we can cope with things in a way that maybe we thought we would not be able to. One of the distinctions that is sometimes made between PTSD and severe stress is that of resilience. These themes are explored in Table 10.

**Table 10:** Domain of change.

Items	Percentage (frequencies)		*M*	*SD*
	Low scores (1–3)	Medium to high scores (4–7)		
*Contribution.* I feel I made a meaningful contribution.	6.6 (7/106) *9.4 (8/85)*	93.4 (99/106) *90.6 (77/85)*	5.63 *5.51*	1.35 *1.32*
*Resilience.* I feel I’ve developed resilience.	1.9 (2/106) *5.9 (5/85)*	98.1 (104/106) *94.1 (80/85)*	5.87 *5.71*	1.16 *1.22*
*Growth.* I have grown as a person.	4.7 (5/106) *9.4 (8/85)*	95.3 (101/106) *90.6 (77/85)*	5.63 *5.46*	1.33 *1.33*
*Life difficulties.* I feel I am able to cope better with life difficulties.	20.8 (22/106) *16.7 (14/84)*	79.2 (84/106) *83.3 (70/84)*	4.85 *4.88*	1.76 *1.62*
*Self-compassion.* I have been able to be self-compassionate during the pandemic.	29.2 (31/106) *25.9 (22/85)*	70.8 (75/106) *74.1 (63/85)*	4.45 *4.54*	1.62 *1.63*
*Close personal relationships.* I have developed closer personal relationships.	35.8 (38/106) *34.5 (29/84)*	64.2 (68/106) *65.5 (55/84)*	4.24 *4.24*	1.86 *1.87*
*Comparisons.* I feel I have coped as well as my work colleagues.	17 (18/106) *16.5 (14/85)*	83 (88/106) *83.5 (71/85)*	4.89 *4.98*	1.56 *1.51*
Negative experiences				
*Difficult personal relationships.* I feel my relationships with others have become more difficult.	48.1 (51/106) *40.5 (34/84)*	51.9 (55/106) *59.5 (50/84)*	3.57 *4.13*	1.96 *1.84*

Top is Portuguese sample. Below italics is UK sample.

#### Contribution

Over and above issues of social support, the feeling that one can make a meaningful contribution is very much linked to well-being (Gilbert, 1984; Gilbert and Simos, 2022). The scores indicate the vast majority (over 90%) of HCPs scored high on their feelings of being able to contribute.

#### Resilience

Importantly, nearly 100% of people thought they had become more resilient which again indicates that, although these are highly traumatic events and some respondents may have had posttraumatic symptoms for a while, their own reflections are ones of developing resilience.

#### Growth

Crucial to posttraumatic growth is obviously the idea that one has ‘grown as a person’ and not just become resilient. This view was endorsed by over 90% of HCPs.

#### Life difficulties

Linked to resilience and growth, 80% of HCPs felt they were better able to cope with life difficulties. In an informal discussion with one of the authors, one colleague noted: ‘once you go through that, you know you can come through most things’.

#### Self-compassion

The ability to be self-compassionate, which is different from being self-reassuring, is linked to coping and well-being. Over 70% of respondents felt they were able to be self-compassionate in the face of the pandemic. However, that does mean nearly 30% seemed to struggle with being self-compassionate. This may, therefore, become a target for helping people when facing these kinds of crises.

#### Close personal relationships

HCPs also acknowledged they had developed close and personal relationships. Anecdotal evidence suggests that some had become more sensitive to the fragilities of life and how to value relationships, although we have no data on this.

#### Comparisons

We often compare ourselves to others and, if unfavourable, that can be a source of self-criticism. However, over 80% of HCPs felt they had coped as well as other colleagues.

#### Difficult personal relationships

The demands of time, the stresses HCPs were under and how these influenced their relationships with others were not without costs – around 55% felt that their personal relationships had become more difficult. Spending longer periods away from family and having to isolate at times put stresses on relationships, although we have no detailed data on this.

### Exploration of standard measures

Tables 11–14 provide data on the four standard measures used in the study.

**Table 11:** Trauma (IES-R; Weiss and Marmar, 1997).

Scale	*M*	SD	Cronbach’s *α*
Total	22.36* 39.52*	19.22 *11.31*	0.96 *0.90*
Intrusion	7.51* 15.18*	7.23 *4.31*	0.93 *0.73*
Avoidance and numbing	8.39* 13.79*	7.49 *4.45*	0.91 *0.78*
Hyperarousal	6.46* 10.55*	5.58 *3.62*	0.88 *0.71*

Top is Portuguese sample. Below italics is UK sample.

*Indicates a significant difference between the Portuguese and UK data on that item, according to an independent samples *t*-test and Mann–Whitney test.

**Table 12:** Social safeness and Pleasure scale.

Scale	*M*	*SD*	Cronbach’s *α*
Total	39.81 *42.35*	9.31 *12.36*	0.94 *0.97*

Top is Portuguese sample. Below italics is UK sample.

**Table 13:** Posttraumatic growth inventory.

Scale	*M*	*SD*	Cronbach’s *α*
Total	*50.60*	*15.73*	*0.90*
Personal strength	*7.72*	*2.75*	*0.43*
New possibilities	*13.56*	*4.44*	*0.71*
Improved relationships	*16.56*	*5.46*	*0.70*
Spiritual growth	*4.91*	*2.29*	*0.60*
Appreciation for life	*7.85*	*2.91*	*0.58*

This data is only available for the UK sample.

**Table 14:** Shirom-Melamed burnout measure.

Scale	*M* (individual scores)	*SD* (individual scores)	Cronbach’s α
Total scale	3.88 *3.65*	1.40 *0.91*	0.96 *0.93*
Physical fatigue	4.61 *4.16*	1.57 *1.11*	0.93 *0.87*
Cognitive weariness	3.86 *3.53*	1.71 *1.00*	0.97 *0.94*
Emotional exhaustion	2.45 *2.84*	1.27 *1.01*	0.88 *0.86*

Top is Portuguese sample. Below italics is UK sample.

Most research investigating the experiences of HCPs involved in COVID-19 contexts focuses on trauma. Table 11 provides the means and standard deviations for trauma (IES-R) in the Portuguese and UK samples.

Scores of 33 and above suggest a PTSD clinical diagnosis (Creamer et al., 2003) and 88 is the highest possible score. The UK participants’ mean total scores were high (39.52) compared to the Portuguese participants’ (22.36). Despite variation, some studies show a similar mean score to the UK data. For example, Nie et al. (2020) showed a mean score of 39.8 for frontline nurses regarding the impact of the pandemic, and Couper et al.’s (2022) study reported a mean of 32.2 during the first wave of COVID-19 with a sample of UK nurses and midwives. The large difference between the samples’ scores might reflect the demographic data on the healthcare workplace, as 84.7% of the UK sample worked primarily in an ICU compared to only 11.3% of the Portuguese sample. A higher number of UK participants also reported they had contracted COVID-19 and lost colleagues to the virus compared with the sample in Portugal. Moreover, the mean scores for the Portuguese sample seem to be similar to other studies where data was collected after the height of the pandemic (Figueiredo et al., 2024) and with non-COVID-19 samples (King et al., 2009; Lee et al., 2018).

Table 12 provides the data for the Social Safeness and Pleasure scale, which measures the degree to which people feel generally secure in their relationships, with a sense of belonging, connectedness, being cared for, wanted and valued (Gilbert et al., 2009). There is now good evidence that this is significantly related to mental health measures and well-being (Armstrong et al., 2020). The scores are consistent with other studies, indicating that HCPs felt as socially connected and safe as the general population (Basran et al., 2019; Kelly et al., 2012). The two samples are equivalent in terms of their sense of social safeness.

The Posttraumatic Growth Inventory data is shown in Table 13. As mentioned, this scale was only administered to UK participants. The highest possible score is 105. Mazor et al. (2016) suggests scores of 46 and above represent medium to high posttraumatic growth (PTG) levels. This indicates that many of the UK respondents have experienced high levels of posttraumatic growth, which aligns with similar PTGI mean scores reported by HCPs in other pandemic-related studies (e.g., Prekazi et al., 2021; Sarıalioğlu et al., 2022; Kalaitzaki et al., 2022; Kapur et al., 2022).

Burnout is explored in Table 14. A number of studies have indicated that burnout is a risk when healthcare professionals are subjected to this degree of distress for long periods and when under pressure because of staff shortages and other factors.

This data reflects the burnout levels of the HCPs within the month leading up to study completion. The maximum individual mean score is 7. Lundgren-Nilsson et al. (2012), Gerber et al. (2018) and Schilling et al. (2019) reported clinically significant burnout as a score of ≥ 4.4. This therefore shows that the UK (M = 3.65), and Portuguese (M = 3.88) samples do not have clinical burnout. The Portuguese sample show clinically significant physical fatigue (M = 4.61) whereas the UK sample do not (M = 4.16).

### Exploring the relationship between trauma and tragedy

Tables 15–19 provide the Spearman’s rank correlations between variables linked to fear-based trauma and variables linked to tragedy-based trauma in both populations, scored when self-report measures were completed (i.e., the ‘do’ data). The tragedy-based trauma data was based on the question: ‘[do you] feel overwhelmed with sadness for the tragedy of pandemic deaths you experienced?’ This item is key to the experience of tragedy. We deliberately used the term *overwhelmed* to try to measure people’s intensity of sadness. To see how this links to symptoms and experiences of fear-based trauma as measured in standard trauma measures, we use the IES-R (Weiss and Marmar, 1997). The relationship between tragedy-based and fear-based trauma is explored in Table 15.

**Table 15:** The relationships of tragedy and trauma.

Tragedy	Trauma total mean score (IES-R)	Avoidance and numbing (IES-R)	Intrusion (IES-R)	Hyperarousal (IES-R)
Tragedy	0.27** −0.02	0.21* 0.01	0.34** −0.03	0.22* −0.04

Top is Portuguese sample. Below italics is UK sample.

*Significant at 0.05 level; **Significant at 0.01 level.

**Table 16:** Emotions in relationship to trauma and tragedy.

Emotion	Tragedy	Trauma (IES-R)
Anxiety	0.37** 0.54**	0.36** 0.06
Anger	0.17 *0.17*	0.18 0.31**
Sadness	0.42** 0.54**	0.46** 0.13
Depression	0.38** 0.38**	0.44** 0.07

Top is Portuguese sample. Below italics is UK sample.

**Significant at 0.01 level.

**Table 17:** Tragedy and trauma in relation to emotions associated with flashbacks.

Emotion	Tragedy	Trauma (IES-R)
Sadness and grief	0.36** 0.55**	0.51** 0.01
Anxiety	0.38** 0.51**	0.47** 0.10
Anger	0.09 0.26*	0.28** 0.19

Top is Portuguese sample. Below italics is UK sample.

*Significant at 0.05 level; **Significant at 0.01 level.

**Table 18:** Compassion from others.

Compassionate support	Tragedy	Trauma (IES-R)	Social safeness (SSPS)	Posttraumatic growth (PTGI)	Burnout (SMBM)	Anxiety	Anger	Sadness	Depression
Being understood	0.17 0.41**	0.03 *0.05*	0.14 0.33**	NA 0.38**	0.01 *0.18*	−0.13 *0.11*	−0.14 *0.01*	0.05 0.25*	0.01 *0.18*
Family support	0.16 0.43**	−0.11 *0.20*	0.48** 0.35**	NA 0.51**	−0.14 *0.05*	0.06 0.36**	−0.09 *0.14*	−0.04 0.33**	0.00 0.23*
Colleagues support	0.12 0.32**	−0.13 *0.20*	0.34** 0.25*	NA 0.39**	−0.25* 0.04	−0.08 *0.14*	0.10 0.22*	0.00 0.25*	−0.07 *0.16*
Openness	−0.01 0.24*	0.00 *0.01*	0.29** 0.23*	NA 0.21*	−0.09 *0.13*	−0.17 *0.11*	−0.10 *−0.05*	−0.08 *0.12*	−0.04 *0.18*
Turning to others	0.07 0.28**	0.14 *−0.06*	0.41** 0.26*	NA 0.25*	−0.12 *0.07*	−0.04 *0.17*	−0.12 *−0.07*	−0.04 *0.13*	0.02 *0.18*

Top is Portuguese sample. Below italics is UK sample.

*Significant at 0.05 level; **Significant at 0.01 level.

**Table 19:** Posttraumatic growth, social safeness and burnout.

Measure	Tragedy	Trauma (IES-R)	Posttraumatic growth (PTGI)	Social safeness (SSPS)
Tragedy	–	–	–	–
Trauma (IES-R)	0.27** −0.02	–	–	–
Posttraumatic growth (PTGI)	NA 0.44**	NA *0.16*	–	–
Social safeness (SSPS)	−0.02 0.37**	−0.24* 0.23*	NA 0.39**	–
Burnout (SMBM)	0.22* 0.11	0.52** −0.15	NA *0.21*	−0.37** 0.01

Top is Portuguese sample. Below italics is UK sample.

*Significant at 0.05 level; **Significant at 0.01 level.

The British sample scored highly on the trauma scale at (M = 39.52), in other words there was significant endorsement of PTSD symptoms. Despite this high rate of symptoms, in the UK sample, there is no correlation with this and the endorsement of feeling overwhelmed (which we labelled as tragedy). This would indicate that fear-based and tragedy-based trauma, or at least the concept and use of these words, are associated with different experiences of distress. The small but significant positive correlation between fear-based and tragedy-based trauma shown for the Portuguese sample perhaps reflects the vastly low proportion of those HCPs working in an ICU compared to the UK sample. ICU staff, typically, have more pre-pandemic experience of witnessing distressing cases, i.e., patients dying, compared to HCPs in general and community wards, thus possibly explaining the increased sadness and sense of feeling overwhelmed in the Portuguese participants. To investigate this further we looked at the pattern of emotions associated with fear-based and tragedy-based trauma.

Table 16 provides correlations for the emotions that HCPs felt about the pandemic in general (given in Table 1) with fear-based and tragedy-based trauma.

This analysis also revealed different patterns of emotions associated with the experience of fear-based vs tragedy-based trauma. For the UK data, feeling overwhelmed by the tragedy is linked to anxiety, sadness and depression, but the same emotions were not associated with fear-based trauma (apart from a small correlation with anger). Again, this indicates these are different types of experience.

Table 17 provides correlations for the emotions associated with flashbacks (given in Table 2) with tragedy-based and fear-based trauma.

Given that flashbacks are very common when going through traumatic events, we were interested in the emotions that textured flashbacks in relation to fear-based and tragedy-based trauma. Surprisingly perhaps, in the UK sample, the standard trauma measure (tapping into fear-based trauma) was not linked to the emotions texturing flashbacks whereas tragedy-based trauma was. Especially notable is that sadness and grief were strongly correlated with conceptualising their experiences of tragedy-based trauma, as well as anxiety and to a lesser degree, anger.

Table 18 shows correlations between tragedy-based and fear-based trauma and compassionate support from others such as feeling understood, family and colleagues’ support and being able to turn to others.

Table 18 indicates that the symptoms of fear-based trauma were not affected by the degree to which people experienced being understood, family support, colleagues’ support, openness and being able to turn to others. We were surprised by this, but again it may mean fear-based trauma is not the appropriate experience in these stressful contexts. When individuals were asked to consider their experiences relating to the concept of tragedy-based trauma however, they did find these sources of support facilitated their ability be aware of, and tolerate feelings of, overwhelming distress—supporting the importance of distinguishing experiences of tragedy-based trauma from experiences of fear-based trauma. However, this was only for the UK sample (mostly frontline workers) and not for the Portuguese sample (mostly community workers). These variables (being understood, family support, colleagues’ support, openness and being able to turn to others) were important for social safeness in both the Portuguese and UK samples, interestingly slightly more so for the Portuguese sample.

Regarding the measure of posttraumatic growth, our data suggests the support people gain from family and colleagues, feeling understood and being open to receiving help are significantly related to this type of change and personal development. This is seen in existing research (Scrignaro et al., 2011). These experiences contributed to a sense of general social safeness and connectedness. Interesting, too, is that these social support factors were not related to burnout, indicating they may operate separately from the challenges of the work itself. Wei et al. (2024) found that secure attachment histories enabled nurses to engage and tolerate painful feelings linked to death. Regarding specific emotions, small variations link to these variables, for example sadness and the ability to tolerate sadness are linked to various forms of support. It is an area requiring further research.

Table 19 explores tragedy-based trauma and fear-based trauma in relation to posttraumatic growth, social safeness and burnout.

Table 19 shows that fear-based trauma and social safeness are significantly negatively correlated in the Portuguese sample whereas in the UK sample, they are significantly positively correlated. While the latter result is less typical, it may reflect the difference between samples regarding the participants’ primary workplaces. As referenced earlier when discussing Table 11, 84.7% of UK HCPs and only 11.3% Portuguese HCPs worked in an ICU. ICUs are known for nurturing greater connectedness and camaraderie between staff due to the high-pressure nature of the workplace (Defilippis et al., 2020; Mealer et al., 2012; Baran et al., 2023; Walsh, 2020; Montgomery et al., 2021) in comparison with community and general wards, possibly explaining the difference between each country’s results.

### Qualitative data from the multicomponent survey of experiences of tragedy-based and fear-based trauma

We invited people to write about and reflect on their experiences in their own way by posing four open-ended questions. The number of participants who chose to answer these questions was very inconsistent, with as low as 34% of all participants answering one of them. Hence, we only comment briefly on some of the themes that emerged, most of which are complementary to the quantitative data above.

#### What was the most difficult aspect of dealing with people outside of your working environment?

Common responses included social distancing, fear of infecting others, dealing with others’ anxiety about being infected (by the HCPs), and the irresponsibility and ignorance of people regarding following COVID-19 restrictions.

#### As a result of what you have been through, is there anything else [excluding emotions discussed] you would like help with?

Many HCPs reported they would like help with anxiety and depression, as well as wanting more help from their organisation, including increased time off, staff, access to resources, guidance, recognition of their service and psychological support.

#### Anything else you found helpful [excluding friends, family, colleagues, professional training, organisational support]?

Participants found a range of activities helpful for coping such as physical exercise, meditation, psychological therapy and support, free time, talking to others, and artistic hobbies (e.g., painting, playing music). One internal resource mentioned by a few Portuguese participants was developing their own resilience.

#### What did you find helpful [about completing the questionnaire]?

The majority of participants provided positive feedback, explaining that the questionnaire enabled useful reflection and insight into their experience as a COVID-19 HCP and their personal growth. A small number of negative comments referred to finding the measure difficult to complete due to reliving negative past experiences or described the survey as too long.

## Discussion

As noted in our introduction, the American Psychological Association (2024) defines trauma as: “*… an emotional response to a terrible event like an accident, crime, natural disaster, physical or emotional abuse, neglect, experiencing or witnessing violence, death of a loved one, war, and more.”* However, these are very different events that will be textured and experienced with different patterns of motives, emotions, cognitions, experiences of the self, others and so forth. While there has been considerable research on reactions, impacts and ‘symptoms’ arising from traumatic events—particularly the big three of hyperarousal, re-experiencing (flashbacks) and numbing—less research has focused on what we call the textures of experience. For example, trauma arising from a rape versus a war injury will texture experience differently compared to trauma arising from trying to save the lives of people who ultimately die. In addition, while for some traumas fear, anger and issues of safety can dominate the experience, loss and grief can also be very important textures. We suggest that these experiences can be captured with concepts of ‘tragedy’ and that can be an important way of languaging certain types of trauma. As noted in the introduction, one of the authors (PG) noted quite major shifts in how trauma was narrated when explored from the point of view of personal tragedy *as well* as fear and loss of safety. Hence, this study developed a survey to tap into possible dimensions of tragedy, a concept that has a long history in the way humans have conceptualised and related to the realities of decay, disease and death.

The motive to address the suffering of life is called compassion, and the courage and wisdom of compassion helps us face the harsh realities of life (Di Bello et al., 2021; Davidson and Harrington, 2002; Gilbert, 2009, 2020; Ricard, 2015; Seppälä et al., 2017). It is well recognised that compassionate caring for those in pain and distress can be distressing to the provider (Behan and Kelly, 2025; Sabin-Farrell and Turpin, 2003; Smiechowski et al., 2021; Shaw and Kelly, 2024). This is particularly important with clinicians working with dying patients. This also relates to how we think about and conceptualise our own ‘trauma-suffering’ experiences as well as processing, narrating and sharing what is happening to other people. The motive of compassion brings us face to face with the tragedies of life and so we explore this in a socially very traumatic and tragic situation which was COVID-19. Although we did not set out with predetermined ideas of how our data would cluster, reflecting on responses suggested five basic clusters of themes which we have presented in five sections.

Section 1: *Self-focused distress*. In regard to the personal experiences of distress, such as anxiety, sadness, depression and flashbacks, the data is very much in line with other studies (Greene et al., 2021). However, sadness is not typically measured in these contexts or is only touched on and can be confused with depression (Horwitz and Wakefield, 2007). Our data suggests a very high frequency of experiences of sadness and tearfulness. Sadness was also one of the main emotions that textured flashbacks.

Opening up to a wider spectrum of experiences revealed high endorsement of multiple experiences including poor sleep and bad dreams, emotional and physical exhaustion. Again, this is in line with other studies using different methodologies (Einvik et al., 2021; Newman et al., 2022). There were also some unique experiences related to the difficulties of wearing PPE. For example, it was very hot, unpleasant to wear and took time to robe-up, making toilet breaks difficult and being off the clinical area for longer than they would like. Masks are commonly seen to interfere with communications such as being unable to facially express care, friendliness or reassurance. HCPs could only be seen through a visa/goggles, making it hard to recognise staff as everyone looked the same. HCPs developed pressure sores on their faces from the masks. Patients could not easily identify who each HCP was, making it difficult for HCPs to provide patient-centred care. Having to always wear gloves meant HCPS felt unable to provide ‘a human touch,’ such as holding a patient’s hand as they died. As these realities of infection control prevented HCPs relating to others in the way they wanted and provided a much disconnected relationship, one could see this as a form of moral trauma but, again, clearly linked to tragedy rather than fear.

A crucial element in coping with compassionate care in this context is how people support and evaluate themselves as they go about their activities. High levels of self-criticism, for example, are associated with mental health difficulties (Werner et al., 2019). Linetsky et al. (2024) found that in midwives, PTSD symptoms were significantly linked with self-criticism. Importantly, it is the harshness and what is called self-attacking forms of self-criticism (Gilbert et al., 2004) that are particularly problematic (Gilbert, 2022b; Whelton and Greenberg, 2005). This explains our question about *harsh* self-criticism, not just criticism, as we wanted to make sure respondents knew they were judging the *harshness* of themselves. While HCPs are obviously concerned with performing their clinical tasks appropriately and adequately, this type of self-monitoring can open the potential for self-criticising. Nearly 60% in the Portuguese population and 67% in the UK population endorsed moderate to high levels of harsh self-criticism. The link between self-criticism and the experience of tragedy may be indirect to the extent that blaming the self hides one from recognising the harshness of life and ones’ powerlessness, ‘maybe it could have been different if only…’. Freud (1919) and Bowlby (1980) noted that we sometimes self-blame to avoid experiencing hostility to others such as those we depend on or authority figures like God. The defensive functions of self-criticism are poorly explored.

In contrast, the ability to be self-reassuring was endorsed by just over half the respondents, but the inverse is that 35–40% scored this question in the lower range, indicating a struggle with self-reassurance. This is important because self-criticism and self-reassurance are significantly linked to mental health and coping (Gilbert et al., 2006; Sommers-Spijkerman et al., 2018). While self-compassion is how we are sensitive to and respond to our distress and suffering, self-reassurance has a more positive focus on remembering one’s competencies and abilities. Although they are correlated, they are different processes (Gilbert et al., 2017). Interesting, then, is that regarding the personal change section, we found that feeling one was able to be self-compassionate during the pandemic was endorsed by over 70%. In general, as anticipated, HCPs experienced a complex range of emotions and experiences. These difficulties also extended to self-relating and struggles with self-criticism and being self-reassuring.

Section 2: *Distress for others’ distress*. At the centre of compassion for others is the empathic sensitivity to the distress and needs of the other (Dalai Lama, 1995; Gilbert, 2009, 2020; Mascaro et al., 2020; Seppälä et al., 2017). Our data shows that this is highly represented in our COVID-19 HCPs. The distinction between distress that relates to one’s own source of suffering (such as fear about being infected, exhaustion, wearing PPE) and that which arises from the distress in the other, called ‘empathic distress’, is not straightforward because they blend into each other. Nonetheless, insight into possible domains of empathic distress is key in helping HCPs to cope. Empathic distress by itself without feeling one can take action can increase personal distress (Di Bello et al., 2021; Gilbert et al., 2017; Klimecki and Singer, 2012). The stress of wanting to protect friends and family was particularly noted at nearly 90%. This dropped to some degree over time but was still quite prevalent when respondents were asked how they felt now. Also, nearly 90% experienced empathic distress; distress from witnessing the suffering of patients. From our discussions, HCPs noted that the way people die is also important. Seeing patients die in pain and/or struggling to breathe was particularly stressful (as noted by one of the authors, WK, who worked in a COVID-19 ICU). Half the HCPs in both the Portugal and UK groups acknowledged they were tearful because of what was happening to their patients. Nearly three quarters found it difficult coming to terms with not being able to save their patients. Important for this study, we found that feelings of tragedy and being overwhelmed by the tragedy of COVID-19 affected over 60% of HCPs.

Section 3: *Openness to the compassion and helpfulness from others*. Wei et al. (2024) found that the way nurses empathised and dealt with this death was linked to attachment history. Hence, the nature of distress in compassionate care providers and what supports them is crucial to explore. Often, to be able to cope with entering into a world of suffering and distress, we are dependent on the compassion and support of others. Our data confirms that family support was felt to be crucial to coping. Important, in this regard, is the recognition that not all HCPs had families close by. Outside of the family, colleague and personal relationships were also helpful, but not to the degree that family relationships were. There was also endorsement of negative experiences. For example, between 50 and 60% of respondents noted some personal relationships had become *more* difficult. Some HCPs felt that people pulled away from them out of fear of being infected.

HCPs also rated their professional training as key to coping. Hence, ensuring that people feel adequately trained and clinically supported helps to offset stress and may build self-reassurance. Obviously, feeling insufficiently trained or supported will be stressful and can lead to self-doubt. This is in line with findings by Aiken et al. (2023) who reported that clinicians would prefer *management* to receive interventions aimed at improving patient care than interventions directed at improving their own mental health and wellbeing.

Section 4: *Organisational stress.* When providing compassionate care within organisational settings, the structure of the organisation itself can be a source of stress. Interestingly, just over 50% of respondents did not feel very familiar with ICUs, partly because they had been brought in from other clinical areas. In one hospital, dental nurses, the army, and staff from other clinical areas (outpatients, paediatrics, etc.) were trained to treat COVID patients. A third of participants acknowledged the anxiety of working in a new intensive clinical area. As is commonly found in underfunded healthcare organisations, management did not fare well, for example in the UK sample, 81% felt the service was poorly managed and psychological support was low.

Section 5: *Personal change*. Talking to HCPs suggested that, while it was very stressful at the time, a combination of working in very close teams, having colleagues’ support, and the fact that they were able to save a lot of people, had built a sense of resilience and strength. Crucial was the sense of having made a contribution and over 90% endorsed these feelings. Over 90% thought they had grown as a person and around 80% felt better able to cope with life difficulties. Moreover, over 80% felt they had coped as well as colleagues. Although 50% felt some relationships had become more difficult, taken together, coming through this highly stressful experience had also built a resilient and self-compassionate sense of self. These experiences indicate how different trauma events and experiences are, as well as the contexts in which they occur and are managed. Early life abuse traumas or rape are very different from the traumas where people are choosing to face them, and in caring for others doing likewise. This also highlights the importance of ‘Schwartz rounds’ where HCPs are encouraged to come together to explore their own feelings of suffering in relation to the suffering they are empathically engaged with (Flanagan et al., 2020).

### Did and do

To try to explore the sense of change in HCPs’ experiences, we designed the survey such that people could reflect on what they remember doing or feeling and what they are doing and feeling now. Of course, one cannot assume that memory is always accurate but it offers impressionistic data of change over time. As can be seen from the data, some experiences and symptoms have changed over time, occasionally by over 50% but for other experiences less so. Regarding Table 1 and primary emotions, there has been a substantial drop in anxiety, anger, depression and sadness but even so the rates are still quite high. For example, retrospectively 83.5% of UK participants scored high to medium for anxiety which dropped to 47.2%. Although that is under 50%, it still means that anxiety is quite highly endorsed in this group and we can see similar patterns for the other emotions too.

In Table 2, relating to the nature and content of flashbacks, we see a similar story that there has been some drop in the emotional intensity of the flashbacks but still relatively high numbers of people are experiencing them at the time of the study.

Table 3 explores the spectrum of experiences and here we can see that at the time of the study the distress from the unpredictability of the pandemic had dropped, but in the UK sample was still at over 70%. Notable too is the fact that distress linked to feeling emotionally numb hardly dropped at all.

Table 4 suggests also relatively little change in self-evaluations, indicating that these may be problematic ways of self-relating that are relatively stable and may need specific attention.

From Table 5, regarding the emotions that people want help with, it seems to be ‘finding joy’ that is particularly salient, with nearly half the group seeing this is an issue. Indeed as Fonzo (2018) highlighted, not enough attention is paid to issues of positive affect and the recovery of positive affect after these kinds of traumas. And this is interesting given the data on feeling numb noted above.

Regarding data from Table 6, although many of the items for empathic distress have dropped over time, they are still quite high. For example, even at the time of data collection, empathic distress is 74.1% for the UK sample.

Exploration of the other items in Table 6 show similar processes where change has occurred but nevertheless some areas remain quite high. What we do not know is the degree to which this is standard for people working in these high stress areas or whether scores are elevated as a result of COVID. If it is the former then we require more detailed studies on these different aspects of experiences of working in these clinical areas and how to support and help people because some of these experiences are not only distressing but are affecting quite high numbers.

### Exploring the relationship between fear-based and tragedy-based trauma

We have argued that trauma experiences which are associated with a *sense of traged*y are different to those that are fear-based and need to be included in conceptualising, narrating and working with certain forms of trauma. Our data seems to support this. For example, in Table 15 we show that there is no relationship between tragedy-based trauma and fear-based trauma on the IES-R in the UK sample, and only small correlations in the Portuguese sample. Furthermore, in Table 16 we found that tragedy-based trauma was significantly correlated with anxiety, sadness and depression across both samples, whereas in the UK sample, fear-based trauma showed a smaller correlation with anger. There was a similar finding when we looked at the emotions that textured flashbacks. Here, again, we find that sadness consistently textures tragedy-based trauma but shows no correlation with fear-based trauma in the UK sample.

In Table 18, we see that the degree of support individuals were receiving do not seem to be associated with symptoms of fear-based trauma, but with the ability to experience sadness (particularly empathic distress). Importantly, posttraumatic growth was also linked to the support people received as they were going through this experience, which corroborates findings from the general population during COVID-19 (Xie and Kim, 2022) and HCPs (Mo et al., 2022; or a review, see Henson et al., 2021). Feeling socially safe and connected was also importantly linked to a range of support from others. In terms of emotions, the support of others had small effects on anxiety, anger and distress. Forms of support increase the ability to acknowledge and experience some of these emotions. It may be that close relationships facilitate safeness for open discussion and greater awareness of one’s emotions. This fits with the finding that attachment history enables empathic engagement with client distress (Wei et al., 2024).

Finally, we looked at the relationships between tragedy-based trauma, fear-based trauma, posttraumatic growth, social safeness and burnout (Table 19). From our data, it seems to be consistent that tuning into sadness and thinking about it as tragedy-based trauma was strongly linked to posttraumatic growth and feeling socially safe, whereas symptoms of fear-based trauma were not.

### Summary

The different types of traumatic events are experienced in different ways. Our study was stimulated by considering that while the issue of fear, hypervigilance and flashbacks along with other dimensions of anxiety and safety are central to trauma, conceptualising some traumas as tragedies opens new dimensions of narrating and conceptualising experiences of suffering. Indeed, the experiences of HCPs who choose to engage with suffering are more nuanced and focused on the issues of empathic distress, sadness and grief (Behan and Kelly, 2025).

While exposure therapies and enabling people to ‘feel safe in the body’ (Van der Kolk, 2014) can help with the tolerance of the fear aspects such as the emotional avoidance of trauma, and safety, processing grief can also be an involved process and requires a ‘safe and sharing relationship’ to enable it (Behan and Kelly, 2025; Gilbert H., 2022; Gilbert, 2022a; Harris and Ho, 2023; Harris and Machado, 2017; Horwitz and Wakefield, 2007). The focus of grief can also vary in ways that we have not explored here. For example, grief can involve a recognition that one cannot go back to being the person one was before, particularly if these kinds of traumas have disrupted one’s conceptualisation and model of the world and oneself in it; called the assumptive world (Harris and Ho, 2023). Traumas can significantly disrupt people’s beliefs about the world (Janoff-Bulman, 2010). They can lead some people to lose (for example) their spiritual or religious beliefs, which can also give a sense of now being separate and different from a group they previously identified with (Leo et al., 2021). People can also lose a sense of connectedness from those who have not been through the same experience, increasing the sense of disorientation and separateness. These are also personal tragedies of loss. Hence the general outcome of this study supports the value of further research in the conceptualisation, nature and treatment of experiencing trauma as tragedy.

### Limitations

Due to various issues raised by ethical committees, our original research agenda was delayed. In addition, we were not able to access COVID-19 HCPs directly, despite efforts particularly by one of the authors. For this reason, HCPs were recruited via charity support groups for COVID-19. As time had progressed, we were past the peak of the crisis and therefore chose to explore people’s reflections on what they felt during the peak and what they were feeling at the time of gathering the data. Also, recruiting primarily through these charities might have led to a selection bias that favoured ICU HCPs. Clearly this is not ideal, but nevertheless we think this study provides a useful platform for subsequent research.

More accurate data on the people involved would have been useful but that was difficult to obtain, again indicating improvements for subsequent studies. This study has not looked at gender differences or differences in age and clinical experience which may be important mediators of personal experiences. Some respondents felt the scales were too long and we were too ambitious. We agree with this, although we faced a dilemma because we knew the time window for getting this data was closing. Retrospective data on trauma was not collected using the IES-R (Weiss and Marmar, 1997) and data only represented participants’ experiences at the time they completed the study. This is not consistent with the past (‘Did’) and present (‘Do’) data gathered in the Multicomponent Survey of Experiences of Tragedy-Based and Fear-Based Trauma, and therefore we were unable to assess change scores for these factors.

The present study utilised the survey method which enabled us to explore a diverse range of subtopics. This is a versatile method that allows the gathering of a large amount of data from diverse groups (Aiken et al., 2023; Greene et al., 2021; Newman et al., 2022). Although our survey items were directed by feedback from clinicians and may inspire future studies, the survey remains open and relatively loose. As raised by one reviewer, it is important to note these limitations and to consider the next stages of research. This would be to develop new insights and therefore measures of tragedy-based trauma. Further qualitative research could be used to explore changes in experience over time. For example, as fear becomes less of a focus, grief and the sense of tragedy can become more prominent in experience. Greater clarity about the textures of tragedy-based trauma could help guide clinicians to variations in intervention. For example, exposure to fear and avoided mental events may not be so helpful for tragedy-based work, whereas grief and anger work might. This is one of balance, not ‘either or’ and a research endeavour. Hence, there are many areas for improvement and development for this kind of research. However, we hope it is robust enough to establish some key principles in terms of trying to distinguish between the complexity of people’s personal experiences beyond diagnostic categories. It also highlights that the older concepts of tragedy, with particular focus on issues of loss, sadness and grieving, remain salient areas of research along with those of the fear-based focus of trauma. This links us to how we all have to deal with the harsh realities of disease, decay, and death of ourselves and others.

## Data Availability

The raw data supporting the conclusions of this article will be made available by the authors, without undue reservation.

## Footnotes

1
https://www.bbc.co.uk/news/health-61023908
