# Supplementary material for: Development of a multicomponent survey of experiences of tragedy-based and fear-based trauma in COVID-19 healthcare professionals
Source: Front Psychol. 2026 Jan 27;16:1513067. doi: 10.3389/fpsyg.2025.1513067 (PMC12887893; doi:10.3389/fpsyg.2025.1513067)
Supplement: Supplementary file 1 [file Supplementary_file_1.docx]

**Multicomponent Survey of Experiences of Stress, Tragedy and Trauma**

Thank you for spending the time to help explore the complex experiences of staff directly involved with COVID-19 patients during the pandemic. Your experiences were intense, stressful, and unique. We have tried to capture some of the possible common experiences below. We would be very grateful if you could reflect on your experience as a worker directly involved with the care of COVID-19 patients. Once again thank you so much for your contribution.

**PART I**

Today’s date: …………………… A date working conditions were most stressful for you?....................

Age…… Gender F / M Prefer not to say……

Were you/are you directly engaged with the care of COVID-19 patients? Y / N

Were you/ are you responsible for working directly with individual patients? Y / N

Is ICU your normal place of work? Y / N Years of experience on an ICU ……………

Were/are you redeployed to work with COVID-19 patients? Y / N

If you were redeployed, which area/ward do you work on normally?

…………………………………………………………………………………………..

Profession……………………. Years since qualified …………..

Have you now moved out of working primarily with COVID-19 patients? Y / N

Have you had COVID-19? Y / N If yes, have you been affected by long COVID? Y / N

Have you lost work colleagues due to COVID-19?  Y / N

Do you have supportive family nearby? Y / N

**PART II**

We have set out a series of questions we hope will capture some of your common experiences. These questions ask about the thoughts and feelings you may have had in your COVID-19 caring role.

**Section 1**

**Psychological and physical reactions**

Please rate the items using the following rating scale in terms of how much the question applies to you.

| 1  Not at all | 2 | 3 | 4  Moderately | 5 | 6 | 7  Extremely |
| --- | --- | --- | --- | --- | --- | --- |

**Did/do** Each question offers you an opportunity to note the degree to which you *did* experience what is suggested in the question and also if you still *do* experience it. This will allow us to see how things may have changed for you.

|  | **Fear distress**. Feel anxious about getting COVID-19 yourself and its impact on you personally? | *Did* | 1 | 2 | 3 | 4 | 5 | 6 | 7 |
| --- | --- | --- | --- | --- | --- | --- | --- | --- | --- |
|  |  | *Do* | 1 | 2 | 3 | 4 | 5 | 6 | 7 |
|  | **Protect friend/family distress**. Feel anxious about getting COVID-19 and then passing it on to others including your friends and family? | *Did* | 1 | 2 | 3 | 4 | 5 | 6 | 7 |
|  |  | *Do* | 1 | 2 | 3 | 4 | 5 | 6 | 7 |
|  | **Empathic distress**. Empathy is when we imagine ourselves in another person’s situation and feel with them. To what extent did/do you become distressed yourself because of what was happening to your patients due to COVID-19? | *Did* | 1 | 2 | 3 | 4 | 5 | 6 | 7 |
|  |  | *Do* | 1 | 2 | 3 | 4 | 5 | 6 | 7 |
|  | **Sadness distress**. Experience sadness as a result of what was happening with your patients due to COVID-19? | *Did* | 1 | 2 | 3 | 4 | 5 | 6 | 7 |
|  |  | *Do* | 1 | 2 | 3 | 4 | 5 | 6 | 7 |
|  | **Tearful.** Moved to tears as a result of what was happening with your patients due to COVID-19? | *Did* | 1 | 2 | 3 | 4 | 5 | 6 | 7 |
|  |  | *Do* | 1 | 2 | 3 | 4 | 5 | 6 | 7 |
|  | **Saving patients.** Find it difficult to come to terms with the fact there were patients you were unable to save due to COVID-19? | *Did* | 1 | 2 | 3 | 4 | 5 | 6 | 7 |
|  |  | *Do* | 1 | 2 | 3 | 4 | 5 | 6 | 7 |
|  | **Tragedy.** Feel overwhelmed with sadness for the tragedy of pandemic deaths you experienced? | *Did* | 1 | 2 | 3 | 4 | 5 | 6 | 7 |
|  |  | *Do* | 1 | 2 | 3 | 4 | 5 | 6 | 7 |
|  | **Responsibility distress**. Feel distressed at having to make life and death decisions? | *Did* | 1 | 2 | 3 | 4 | 5 | 6 | 7 |
|  |  | *Do* | 1 | 2 | 3 | 4 | 5 | 6 | 7 |
|  | **Helpless distress**. Feel distressed because you felt/feel unable to prevent people you were caring for from dying or harm? | *Did* | 1 | 2 | 3 | 4 | 5 | 6 | 7 |
|  |  | *Do* | 1 | 2 | 3 | 4 | 5 | 6 | 7 |
|  | **Unpredictability distress**. Feel distressed because it was uncertain how long the pressure from the pandemic would last? | *Did* | 1 | 2 | 3 | 4 | 5 | 6 | 7 |
|  |  | *Do* | 1 | 2 | 3 | 4 | 5 | 6 | 7 |
|  | **Sleep.** Experience sleep difficulties because of working with COVID-19 patients? | *Did* | 1 | 2 | 3 | 4 | 5 | 6 | 7 |
|  |  | *Do* | 1 | 2 | 3 | 4 | 5 | 6 | 7 |
|  | **Bad Dreams.** Have difficult or nightmarish dreams? | *Did* | 1 | 2 | 3 | 4 | 5 | 6 | 7 |
|  |  | *Do* | 1 | 2 | 3 | 4 | 5 | 6 | 7 |
|  | **Emotional exhaustion**. Find yourself becoming emotionally exhausted? | *Did* | 1 | 2 | 3 | 4 | 5 | 6 | 7 |
|  |  | *Do* | 1 | 2 | 3 | 4 | 5 | 6 | 7 |
|  | **Numbness distress**. Find yourself becoming emotionally numb? | *Did* | 1 | 2 | 3 | 4 | 5 | 6 | 7 |
|  |  | *Do* | 1 | 2 | 3 | 4 | 5 | 6 | 7 |
|  | **Physical exhaustion**. Find yourself becoming physically exhausted? | *Did* | 1 | 2 | 3 | 4 | 5 | 6 | 7 |
|  |  | *Do* | 1 | 2 | 3 | 4 | 5 | 6 | 7 |
|  | **PPE.** Find wearing PPE distressing and difficult because it was uncomfortable? | *Did* | 1 | 2 | 3 | 4 | 5 | 6 | 7 |
|  |  | *Do* | 1 | 2 | 3 | 4 | 5 | 6 | 7 |
|  | **PPE.** Find wearing PPE difficult due to it interfering with you relating to patients? | *Did* | 1 | 2 | 3 | 4 | 5 | 6 | 7 |
|  |  | *Do* | 1 | 2 | 3 | 4 | 5 | 6 | 7 |
|  | **Breathing.** Compared to other patient difficulties you have worked with, to what extent did/do you find the specific problems of breathing difficulties due to COVID-19 in patients distressing? | *Did* | 1 | 2 | 3 | 4 | 5 | 6 | 7 |
|  |  | *Do* | 1 | 2 | 3 | 4 | 5 | 6 | 7 |
| **Primary emotions.** To what extent did/do the emotions below describe your primary feelings towards the pandemic? | | | | | | | | | |
| 19. | Anxiety | *Did* | 1 | 2 | 3 | 4 | 5 | 6 | 7 |
|  |  | *Do* | 1 | 2 | 3 | 4 | 5 | 6 | 7 |
| 20. | Anger | *Did* | 1 | 2 | 3 | 4 | 5 | 6 | 7 |
|  |  | *Do* | 1 | 2 | 3 | 4 | 5 | 6 | 7 |
| 21. | Sadness | *Did* | 1 | 2 | 3 | 4 | 5 | 6 | 7 |
|  |  | *Do* | 1 | 2 | 3 | 4 | 5 | 6 | 7 |
| 22. | Depression | *Did* | 1 | 2 | 3 | 4 | 5 | 6 | 7 |
|  |  | *Do* | 1 | 2 | 3 | 4 | 5 | 6 | 7 |

**Section 2- Organisational Stress**

These questions ask you about your thoughts and feelings about the organisation you work/worked in.

Please rate the items using the following rating scale in terms of how much the question applies to you.

| 1  Not at all | 2 | 3 | 4  Moderately | 5 | 6 | 7  Extremely |
| --- | --- | --- | --- | --- | --- | --- |

**Did/do** Some of the questions below offer you an opportunity to note the degree to which you *did* experience what is suggested in the question and also if you still *do* experience it. This will allow us to see how things may have changed for you.

| 1. | **Familiarity.** Were you familiar with the workings of an intensive care unit? | 1 | 2 | 3 | | 4 | | 5 | 6 | 7 |
| --- | --- | --- | --- | --- | --- | --- | --- | --- | --- | --- |
| 2. | **Anxiety over work area**. How anxious were you moving into an intensive care unit? | 1 | 2 | 3 | | 4 | | 5 | 6 | 7 |
|  | **Anger/frustration distress for you**. Feel angry, irritated or frustrated by equipment shortages for you? e.g. PPE | *Did* | 1 | 2 | 3 | | 4 | 5 | 6 | 7 |
|  |  | *Do* | 1 | 2 | 3 | | 4 | 5 | 6 | 7 |
|  | **Anger/frustration distress for others**. Feel angry, irritated or frustrated by equipment shortage for your patients? e.g. ventilators, beds. | *Did* | 1 | 2 | 3 | | 4 | 5 | 6 | 7 |
|  |  | *Do* | 1 | 2 | 3 | | 4 | 5 | 6 | 7 |
|  | **Management.** Feel distressed by the way your service was managed? | *Did* | 1 | 2 | 3 | | 4 | 5 | 6 | 7 |
|  |  | *Do* | 1 | 2 | 3 | | 4 | 5 | 6 | 7 |
|  | **Shift distress.** Feel distressed because of shift patterns? | *Did* | 1 | 2 | 3 | | 4 | 5 | 6 | 7 |
|  |  | *Do* | 1 | 2 | 3 | | 4 | 5 | 6 | 7 |
|  | **Psychological support.** Feel your organisation provided you with psychological support to help you through the crisis? | *Did* | 1 | 2 | 3 | | 4 | 5 | 6 | 7 |
|  |  | *Do* | 1 | 2 | 3 | | 4 | 5 | 6 | 7 |

**Section 3**

**Relationships with others**

These questions ask about your thoughts and feelings regarding your relationship with others.

Please rate the items using the following rating scale in terms of how much the question applies to you.

| 1  Not at all | 2 | 3 | 4  Moderately | 5 | 6 | 7  Extremely |
| --- | --- | --- | --- | --- | --- | --- |

**Did/do** Each question offers you an opportunity to note the degree to which you *did* experience what is suggested in the question and also if you still *do* experience it. This will allow us to see how things may have changed for you.

|  | **Being understood**. Feel other people can understand what you have been through? | *Did* | 1 | 2 | 3 | 4 | 5 | 6 | 7 |
| --- | --- | --- | --- | --- | --- | --- | --- | --- | --- |
|  |  | *Do* | 1 | 2 | 3 | 4 | 5 | 6 | 7 |
|  | **Family support**. Feel you have family and friends who you can turn to for support and help? | *Did* | 1 | 2 | 3 | 4 | 5 | 6 | 7 |
|  |  | *Do* | 1 | 2 | 3 | 4 | 5 | 6 | 7 |
|  | **Colleagues support**. Feel you have work colleagues who you can turn to for support and help? | *Did* | 1 | 2 | 3 | 4 | 5 | 6 | 7 |
|  |  | *Do* | 1 | 2 | 3 | 4 | 5 | 6 | 7 |
|  | **Expectations**. Feel others expect you to cope better with these experiences? | *Did* | 1 | 2 | 3 | 4 | 5 | 6 | 7 |
|  |  | *Do* | 1 | 2 | 3 | 4 | 5 | 6 | 7 |
|  | **Avoidance.** Feel other people have moved away from you in fear of getting infected by you? | *Did* | 1 | 2 | 3 | 4 | 5 | 6 | 7 |
|  |  | *Do* | 1 | 2 | 3 | 4 | 5 | 6 | 7 |
|  | **Openness.** Feel comfortable sharing distressing feelings with others? | *Did* | 1 | 2 | 3 | 4 | 5 | 6 | 7 |
|  |  | *Do* | 1 | 2 | 3 | 4 | 5 | 6 | 7 |
|  | **Turning to others.** Feel comfortable turning to others and accepting their help? | *Did* | 1 | 2 | 3 | 4 | 5 | 6 | 7 |
|  |  | *Do* | 1 | 2 | 3 | 4 | 5 | 6 | 7 |
| 8. What was the most difficult aspect of dealing with people outside of your working environment?................................................................................................................. | | | | | | | | | |

**Section 4**

**Current Mental States**

These questions ask about mental states you may have experienced in your COVID-19 caring role.

Please rate the items using the following rating scale in terms of how much the question applies to you.

| 1  Not at all | 2 | 3 | 4  Moderately | 5 | 6 | 7  Extremely |
| --- | --- | --- | --- | --- | --- | --- |

**Did/do** Some of the questions below offer you an opportunity to note the degree to which you *did* experience what is suggested in the question and also if you still *do* experience it. This will allow us to see how things may have changed for you.

|  | **Flashback frequency.** Get flashbacks or sudden switches in your mental state that take you back to those distressing experiences? | *Did* | 1 | 2 | 3 | 4 | 5 | 6 | 7 |
| --- | --- | --- | --- | --- | --- | --- | --- | --- | --- |
|  |  | *Do* | 1 | 2 | 3 | 4 | 5 | 6 | 7 |
| **Flashback emotion.** If you did/do have flashbacks, to what extent are they associated with feelings of | | | | | | | | | |
| 2. | Sadness and grief? | *Did* | 1 | 2 | 3 | 4 | 5 | 6 | 7 |
|  |  | *Do* | 1 | 2 | 3 | 4 | 5 | 6 | 7 |
| 3. | Anxiety? | *Did* | 1 | 2 | 3 | 4 | 5 | 6 | 7 |
|  |  | *Do* | 1 | 2 | 3 | 4 | 5 | 6 | 7 |
| 4. | Anger? | *Did* | 1 | 2 | 3 | 4 | 5 | 6 | 7 |
|  |  | *Do* | 1 | 2 | 3 | 4 | 5 | 6 | 7 |
| 5. | **Self-criticism**. Find it easy to become harshly self-critical of yourself? | *Did* | 1 | 2 | 3 | 4 | 5 | 6 | 7 |
|  |  | *Do* | 1 | 2 | 3 | 4 | 5 | 6 | 7 |
| 6. | **Self-reassurance.** Find you are able to reassure and support yourself when things are difficult? | *Did* | 1 | 2 | 3 | 4 | 5 | 6 | 7 |
|  |  | *Do* | 1 | 2 | 3 | 4 | 5 | 6 | 7 |

| **Help with.** As a result of what you have been through, to what extent do you find the emotion(s) you most need help with | | | | | | | | |
| --- | --- | --- | --- | --- | --- | --- | --- | --- |
| 7. | are sadness and grief? | 1 | 2 | 3 | 4 | 5 | 6 | 7 |
| 8. | is fear? | 1 | 2 | 3 | 4 | 5 | 6 | 7 |
| 9. | is anger? | 1 | 2 | 3 | 4 | 5 | 6 | 7 |
| 10. | is finding joy? | 1 | 2 | 3 | 4 | 5 | 6 | 7 |
| 11. Anything else you would like help with?  ……………………………………………………………………………………………………………………………………….  ……………………………………………………………………………………………………………………………………….  ………………………………………………………………………………………………………………………………………. | | | | | | | | |
| What has helped you most? | | | | | | | | |
| 12. | Family | 1 | 2 | 3 | 4 | 5 | 6 | 7 |
| 13. | Friends | 1 | 2 | 3 | 4 | 5 | 6 | 7 |
| 14. | Work colleagues | 1 | 2 | 3 | 4 | 5 | 6 | 7 |
| 15. | My professional training | 1 | 2 | 3 | 4 | 5 | 6 | 7 |
| 16. | Organisational support | 1 | 2 | 3 | 4 | 5 | 6 | 7 |
| 17. Anything else you found helpful?  ……………………………………………………………………………………………………………………………………….  ……………………………………………………………………………………………………………………………………….  ………………………………………………………………………………………………………………………………………. | | | | | | | | |

**Section 5**

**Personal Change**

These questions ask about your thoughts and feelings regarding how you have changed as a result of the COVID-19 crisis.

In regards to how you feel at the moment, to what extent do you feel changed by your experiences in the context of the pandemic?

Please rate the items using the following rating scale:

| 1  Not at all | 2 | 3 | 4  Moderately | 5 | 6 | 7  Extremely |
| --- | --- | --- | --- | --- | --- | --- |

| 1. | **Contribution.** I feel I made a meaningful contribution. | 1 | 2 | 3 | 4 | 5 | 6 | 7 |
| --- | --- | --- | --- | --- | --- | --- | --- | --- |
| 2. | **Resilience.** I feel I’ve developed resilience. | 1 | 2 | 3 | 4 | 5 | 6 | 7 |
| 3. | **Growth.** I have grown as a person. | 1 | 2 | 3 | 4 | 5 | 6 | 7 |
| 4. | **Life difficulties.** I feel I am able to cope better with life difficulties. | 1 | 2 | 3 | 4 | 5 | 6 | 7 |
| 5. | **Close personal relationships.** I have developed closer personal relationships. | 1 | 2 | 3 | 4 | 5 | 6 | 7 |
| 6. | **Difficult personal relationships.** I feel my relationships with others have become more difficult. | 1 | 2 | 3 | 4 | 5 | 6 | 7 |
| 7 | **Self-compassion.** I have been able to be self-compassionate during the pandemic. | 1 | 2 | 3 | 4 | 5 | 6 | 7 |
| 8 | **Comparisons.** I feel I have coped as well as my work colleagues. | 1 | 2 | 3 | 4 | 5 | 6 | 7 |

Has answering these questions been helpful to you when thinking about your experience?

YES ___ NO___

What did you find helpful?

............................................................................................................................................................................................................................................................................................................................................................................................................................................
